# Odd-even effects in lead-iodide-based Ruddlesden–Popper 2D perovskites†

**DOI:** 10.1039/d5ta01234a

**Published:** 2025-05-19

**Authors:** Maryam Choghaei, Maximilian Schiffer, Viren Tyagi, Marcello Righetto, Jiaxing Du, Maximilian Buchmüller, Kai Oliver Brinkmann, Geert Brocks, Patrick Görrn, Laura M. Herz, Shuxia Tao, Thomas Riedl, Selina Olthof

**Affiliations:** a Department of Chemistry, University of Cologne Greinstrasse 4-6 50939 Cologne Germany solthof@uni-koeln.de; b Institute of Electronic Devices, University of Wuppertal Rainer-Gruenter-Str. 21 42119 Wuppertal Germany; c Wuppertal Center for Smart Materials & Systems (CM@S), University of Wuppertal 42119 Wuppertal Germany; d Materials Simulation & Modelling, Department of Applied Physics and Science Education, Eindhoven University of Technology 5600 MB Eindhoven Netherlands; e Department of Physics, University of Oxford, Clarendon Laboratory Parks Road Oxford OX1 3PU UK; f Chair of Large Area Optoelectronics, University of Wuppertal Rainer-Gruenter-Str. 21 42119 Wuppertal Germany; g Computational Chemical Physics, Faculty of Science and Technology, MESA+ Institute for Nanotechnology, University of Twente 7500 AE Enschede Netherlands

## Abstract

Two-dimensional (2D) halide perovskites are a versatile material class, exhibiting a layered crystal structure, consisting of inorganic metal–halide sheets separated by organic spacer cations. Unlike their 3D counterparts, 2D perovskites have less strict geometric requirements, allowing for a wider range of molecules to be incorporated. This potentially offers a way to engineer the properties of a 2D perovskite through adequate selection of the organic spacer cations. Our study systematically analyzes the effect of spacer cation length on the electronic and optical properties of Ruddlesden–Popper lead-iodide-based 2D perovskites, using alkylammonium cations of varying chain lengths. Intriguingly, no linear correlation between interlayer distance and the optical gap or valence band position is observed in our measurements. Rather it matters whether the spacer cation contains an odd or even number of carbon atoms in the chain. Notably, these odd-even effects manifest in variations of ionization energy, optical gap as well as charge carrier mobility. Density functional theory calculations reproduce the changes in optical properties, allowing us to identify the underlying mechanism: while even-numbered carbon chains pack efficiently within the organic spacer layer, the shorter odd-numbered chains increase distortions. These distortions lead to variations in the Pb–I–Pb bond angle within the inorganic sheets, resulting in the observed odd-even effect in the (opto-)electronic properties. This understanding will be helpful to make more informed choices regarding the incorporated spacer molecules which can potentially help to enhance performance when integrating such 2D perovskite interlayers into devices.

## Abstract 2

An odd-even parity effect in Ruddlesden–Popper 2D perovskites is demonstrated, where spacer cation chain length governs structural distortions that systematically influence band gap, ionization energy and in-plane carrier mobility.

## Graphical abstract

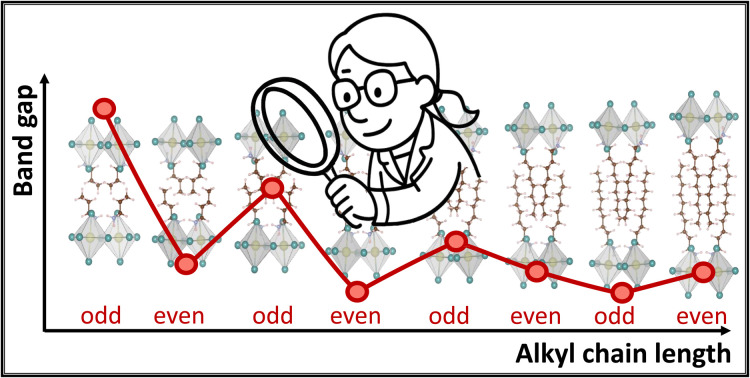

## 1 Introduction

Over the last decade, halide perovskites have emerged as one of the most promising classes of semiconductors, sparking remarkable advancements across multiple applications. The ever-increasing accomplishments of this class of semiconductors are most notable in photovoltaics,^1^ but their applications as light emitters,^2,3^ lasing materials^4^ and photodetectors^5^ hold significant potential. Three-dimensional (3D) perovskites still dominate the field of application, however increasing attention is lately given to two-dimensional (2D) perovskites. Most commonly Ruddlesden–Popper (RP) phases are employed here, which can form when a large hydrophobic mono-amine spacer A′ is either fully 
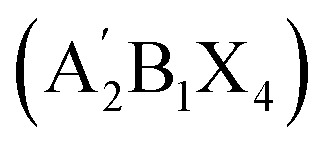
 or partially (
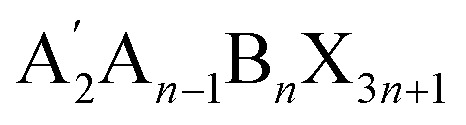
, *i.e.* quasi 2D structure) replacing the small A site cations.^6,7^ The resulting structure consists of alternating hydrophobic sheets of insulating organic spacer cations and *n* sheets of the semiconducting inorganic metal halide [BX_6_]^4−^ octahedra, forming a quantum well structure. Characteristic for the RP structure is a relative shift between adjacent inorganic slabs, enabled by the weak van der Waals interactions between the organic spacers, which distinguishes them from Dion–Jacobson (DJ) phases where no such slipping occurs. Such layered semiconductors exhibit an increased band gap compared to their 3D counterparts, anisotropic charge transport, strongly bound excitons and a high oscillator strength because of the dielectric confinement. They have gained relevance as thin interlayers on top of the 3D perovskite, next to the adjacent charge transport layer, forming a hydrophobic surface layer that seems not to affect the vertical charge transport significantly. Indeed, implementation of such 2D capping layers often proved beneficial for the charge extraction process, which is why a large number of current high-efficiency photovoltaic devices employ such a 2D capping layer.^8,9^ Even though these 2D interlayers are successfully employed in devices and have been shown to increase the efficiency and stability of perovskite solar cells, the exact role of these thin 2D layers remains elusive. In addition to the hydrophobicity, various studies also indicate the passivation of traps at the 3D perovskite surface^8,10^ and improved charge-carrier selectivity^11,12^ due to advantageous band offsets. To further clarify these effects, it is essential to explore how the dimensionality, choice of organic cation and spacing between the inorganic sheets influence key properties such as energetic structure, band alignment and band gap.

Similar to what has been reported for 3D perovskite, the choice of metal on the B-site and the halide on the X-site can be expected to matter for the band structure and therefore the energetic position of the valence band (VB) and conduction band (CB).^13^ In contrast, it is usually assumed that the organic cation does not significantly contribute to the density of states at the band edges and therefore has no direct influence on the electronic structure and optical properties. However, experimental^14–17^ as well as theoretical^18–20^ studies indicate that the integration of the spacer cations can lead to octahedral tilting within the inorganic sheet or variations in the metal–halide bond length. Those distortions could affect the wave function overlap and thereby change the band gap. An extensive review of the effect that spacer cations can have on the formation, optoelectronic properties, device performance and stability can be found in the review article ref. 21.

The number of possible aliphatic and aromatic organic cations that can be incorporated into 2D perovskite structures is huge,^22^ and it is important to gain an understanding of how much and in which way the choice of molecule can affect the overall material properties and whether they can be exploited to fine-tune the optoelectronic characteristic. The first optical study on lead-iodide-based 2D halide perovskites was published in 1989 by Ishihara *et al.* using *n*-decylammonium (C_10_H_21_NH_3_^+^) as a spacer cation. They found an excitonic feature at 2.4 eV with a high exciton binding energy of 370 meV. They attributed the strong Coulomb interaction to the spatial confinement within the inorganic sheets and the dielectric confinement by the pronounced difference in relative permittivity between these inorganic sheets and the surrounding organic cations.^23^

In 2D perovskite structures, the size of the spacer cation tunes the distance between the inorganic sheets which raises the question of whether or not the interlayer distance has a noticeable effect on the optical properties. Theoretical studies have suggested that for shorter organic cations the confinement is not fully effective and a small residual communication between the inorganic planes prevails,^20^ which will effectively lower the band gap. Indeed, interlayer coupling was experimentally observed for 2D perovskites containing the rather short organic cation ethanolammonium iodide.^24^ In contrast, a study of spacer cations of different lengths, ranging from C_4_H_9_NH_3_^+^ to C_12_H_25_NH_3_^+^, observed barely any changes in optical properties, leading to the conclusion that the interaction between the inorganic sheets is overall rather weak.^25^ Indeed, the optical properties reported for vastly different organic cations in lead-iodide-based 2D perovskite mostly match rather well with reported photoluminescence (PL) peaks around 2.3 ± 0.1 eV,^6,14,24^ similar to what Ishihara *et al.* reported for *n*-decylammonium in 1989.^23^

While the band gap of lead-iodide-based 2D perovskite is widely studied and typically agrees within the error margin of ±0.1 eV, the understanding of changes regarding energy level positions is lacking. Current literature reports disagree on whether both the valence and conduction bands are playing a role in increasing the band gap. If both energy levels are affected and move away from the center of the band gap, then this increase in ionization energy (IE) and decrease in electron affinity (EA) would result in a straddling junction (so-called type I) when combining a 2D layer with a 3D one. This has indeed been experimentally observed using UV photoelectron spectroscopy (UPS) when comparing either individually measured 3D and 2D perovskites^26–28^ or 3D perovskites passivated with the organic spacer cations.^29^ However, a similar number of studies find that the IE is barely affected or even decreases for the 2D perovskites^30–32^ or upon surface treatment,^33,34^ resulting in the prediction of a type II staggered junction between 3D and 2D layers. While a straddling junction would lead to a charge-blocking behavior for electrons as well as holes at this 3D/2D interface, a staggered junction would allow for efficient hole extraction (see ESI Fig. S1† for corresponding schematic energy level diagrams).

In this current work, we systematically study the effect of organic spacer cation length, and therefore interplane separation, on the optical, electronic and electrical properties of RP-type lead-iodide 2D perovskites with *n* = 1. We selected a series of non-conjugated alkyl spacers and varied the length, starting from the 3D-forming methylammonium (CH_3_NH_3_^+^) cation containing only one carbon atom, and increasing up to *n*-decylammonium (C_10_H_24_N^+^) containing 10 carbon atoms in the alkyl chain, see Table 1. The quality of the films was characterized using scanning electron microscopy (SEM) and further more X-ray diffraction (XRD) was used which revealed that not all spacer cations form phase-pure 2D layers. Using UPS to analyze the electronic structure, only minor variations in IE were found with spacer length, and notably, these values are similar to what is observed also for the 3D MAPbI_3_. In optical reflectance measurements as well as photoluminescence spectroscopy an intriguing odd-even effect is observed, with even-numbered materials all exhibiting the same optical gap, while some of the odd-numbered compounds have larger band gaps. Density functional theory calculations confirm this trend and link it to variations in the Pb–I–Pb bond angle in the inorganic sheet caused by the more inefficient packing of the odd-numbered spacer molecules. Finally, measurements by optical-pump terahertz-probe (OPTP) spectroscopy are used to study whether the packing also affects the in-plane transport of charge carriers in these films. Here, the odd-even effect is also revealed, leading to significantly higher charge-carrier mobilities for the even-numbered spacer cations compared to the odd-numbered varieties.

**Table 1:** Chemical structures and naming convention of organic spacer cations used in the perovskite materials under investigation here

Organic spacer cation	Label	Molecular structure
Methylammonium	C1	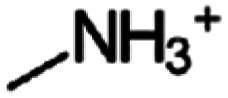
Ethylammonium	C2	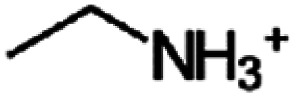
*n*-Propylammonium	C3	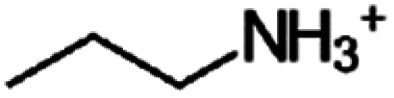
*n*-Butylammonium	C4	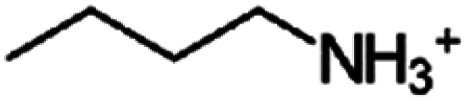
*n*-Pentylammonium	C5	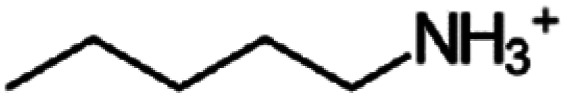
*n*-Hexylammonium	C6	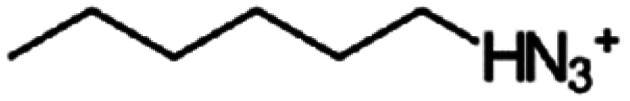
*n*-Hepthylammonium	C7	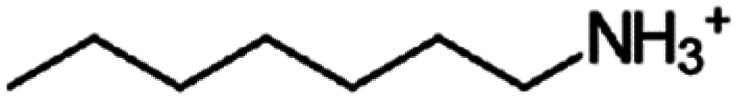
*n*-Octylammonium	C8	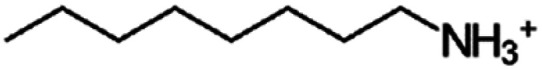
*n*-Nonylammonium	C9	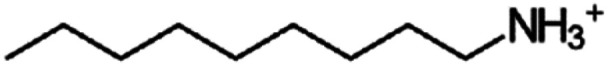
*n*-Decylammonium	C10	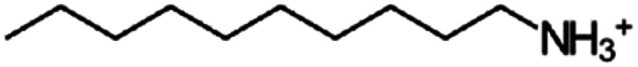

## 2 Materials and methods

### 2.1 Materials

As substrates, indium tin oxide (ITO) coated glass, glass (Schott AG), and silicon substrates (MicroChemicals GmbH) were used, which were consecutively cleaned in deionized (DI) water, ethanol and isopropyl alcohol (IPA). The perovskite films were either deposited directly onto the ITO, or an additional layer of approximately 30 nm of PEDOT:PSS (Clevios 4083, Heraeus) was inserted in between. For the study of 2D perovskites, different cation alkylammonium iodide salts (C_*x*_H_2*x*+1_NH_3_I), as listed in Table 1, are explored. Here the carbon chain lengths range from *x* = 1 (methylammonium iodide, labeled as C1) to *x* = 10 (*n*-decylammonium iodide, labeled as C10). The halide salts corresponding to C2–C6 and C8 were purchased from TCI, while *n*-hepthylammonium iodide (C7), *n*-nonylammonium iodide (C9), and *n*-decylammonium iodide (C10), were synthesized in-house. The methylammonium iodide (MA, labeled as C1) for the preparation of the 3D reference material was purchased from Great Cell Solar Materials.

### 2.2 Film fabrication

The 2D perovskite precursor solutions were prepared by dissolving lead iodide (TCI) and the organic cation salts (molar ratio of PbI_2_/(C*x*)I = 1 : 2) in dimethylformamide (DMF; Sigma-Aldrich). The precursor was heated and stirred at 50 °C for 3 h before spin coating. DMF was used as solvent since we noticed that DMSO led to the formation of metallic lead in the samples, as observed by XPS measurements (see ESI Fig. S2†). Using these solutions, the thin films were deposited by spin-coating at 3000 rpm for 40 s (3 s ramp), followed by thermal annealing at 100 °C for 10 min. All ink and sample preparation were done in an N_2_ atmosphere. The MAPbI_3_ reference sample was prepared by dissolving MAI and PbI_2_ in DMF in a 1 : 1 molar ratio. The solution was deposited on the substrate by spin-coating the precursor at 5000 rpm for 30 s (2 s ramp). 5 s after the beginning of the spin-coating, 200 μL of chlorobenzene as antisolvent was dripped on the spinning substrate. The resulting thin film was annealed at 100 °C for 1 h.

As indicated above, either ITO or PEDOT:PSS was used as a substrate for the 2D perovskites. During the initial film optimization, we noticed that the layer formation, particularly the wetting of the perovskite precursor ink and thereby the homogeneity of the film, was strongly affected by the choice of substrate. Best film formation was achieved by using PEDOT:PSS for the shorter chain-based perovskite films (based on C3 and C4) while ITO was used for C5–C10. Notably, all layers show a rather homogenous and complete coverage, as can be seen in the top-view SEM images in Fig. S3.† It should be noted that nonetheless, with higher C*x* the films become less homogenous, in particular for C9 and C10-based perovskites. The hydrophobicity of the larger cations disrupts the wetting properties of the solution, leading to inferior coverage of the perovskite film. This could be partially circumvented by changing the concentration from 0.25 M to 0.75 M, however, variations in film thickness throughout the sample remained a challenge here, as illustrated in ESI Fig. S4.†

### 2.3 XRD

The crystallographic properties of the perovskite films were analyzed using X-ray diffraction (XRD) on a Panalytical Empyrean system with Cu K_α_ radiation (*λ* = 0.1541 nm). The XRD patterns were recorded over a 2*θ* range of 3° to 40° in air at room temperature. The films were scanned continuously with a step size of 0.01° and a scanning speed of 0.1° s^−1^. For this measurement, thicker samples with a concentration of 1 M were prepared on glass substrates. Note, as further discussed below, for the investigation of different polymorphs,^35^ for the C3-based perovskite also the lower concentration of 0.125 M was tested.

### 2.4 UV-vis

The samples for UV-vis absorption from diffuse reflectance were spin-coated from 1 M precursor solutions on glass substrates and measurements were conducted at room temperature with a fiber spectrometer (Ocean Optics QE Pro Series) operating between 300–1100 nm. The detection system was equipped with an integrating sphere, while a white light source (Energetiq EQ-99X LDLS) was used for illumination. The absorption coefficient *α* is derived from the Kubelka–Munk relation *α*/*S* = (1 − *R*)^2^/2*R* where *S* is a constant arbitrary scattering parameter and *R* is the diffuse reflectance.^36^ For C3, to guarantee the formation of the desired (C3)_2_PbI_4_ polymorph, a lower precursor concentration had to be used, resulting in layers thinner than required for the Kubelka–Munk relation to be valid. For these, *α* has been determined by a transmittance/reflectance measurement in the same setup.

### 2.5 PL measurement

For photoluminescence (PL) measurements similar samples as for the UV-vis analysis have been used. A diode-pumped, frequency-tripled solid-state laser (*λ* = 355 nm, Roithner RLTUVL-355-10-10) with a power density *P* = 0.64 W cm^−2^ was used. The PL was fiber-coupled into a monochromator (Princeton Instruments, Acton SP2500, gratings: 300 lines per mm) and detected by a thermoelectrically cooled charge-coupled device camera (Princeton Instruments, PIXIS-100). Temperature-dependent measurements were performed by regulating the temperature *via* a 65 W Peltier module while monitoring the temperature with a PT100 temperature sensor.

### 2.6 Ultra-violet photoelectron spectroscopy

UPS measurements were performed using a hemispherical electron energy analyzer (Specs Phoibos 100) to detect the photoelectrons emitted from the sample surface. For excitation, UV light from a monochromated helium discharge lamp (VUV5k, ScientaOmicron) at *hν* = 21.22 eV was used. Perovskite samples for these measurements were prepared on PEDOT:PSS for C3 and C4 and on ITO substrates for C5–C8 using a solution concentration of 0.25 M. This low concentration was used to avoid potential charging of a thicker film during UPS measurements since these 2D perovskites exhibit layer alignment parallel to the substrate and are thus much less vertically conductive than their 3D counterparts. Nonetheless, for C9 and C10 a higher concentration of 0.75 M had to be used to achieve full coverage of the samples, as discussed before.

### 2.7 Terahertz spectroscopy

Optical-pump terahertz-probe (OPTP) transients were acquired using a setup described in detail elsewhere.^37,38^ In brief, a fraction of the fundamental output of an amplified Ti:sapphire laser system (Spectra-Physics Spitfire ACE) – 1.55 eV, 5 kHz repetition rate, 35 fs pulse duration – was used to generate single-cycle THz radiation pulses. THz pulses were generated using a spintronic emitter, a W/Co_40_Fe_40_B_20_/Pt thin film on sapphire, with anti-reflective and high-reflectivity coating layers.^39,40^ For this experiment, the perovskite films were cast on 2 mm thick z-cut quartz substrates *via* spin-coating. Here, the precursor solution concentration and spin-coating speed were carefully optimized to achieve a uniform film thickness of 110–140 nm. Specifically, a 0.3 M solution was spin-coated at 3000 rpm for C3 and C4-based perovskite, 0.3 M at 4000 rpm for C5, 0.27 M at 4000 rpm for C6, 0.28 M at 5000 rpm for C7, 0.26 M at 5000 rpm for C8, and 0.25 M at 4000 rpm for C9. The formation of a C10-based perovskite film of this thickness with sufficient homogeneity was not successful, it was therefore not included in this study. The THz radiation transmitted through the sample was detected using free-space electro-optic sampling (EOS) in a 1 mm thick (110) ZnTe crystal. In OPTP measurements, fractional changes in the THz transmission (0.5–2.5 THz) following the 3.1 eV (400 nm) pulsed photoexcitation were monitored as a function of pump delay time. During OPTP measurements, the THz emission, detection optics and samples were kept under vacuum at pressures below 0.1 mbar.

### 2.8 Density functional theory (DFT) calculations

The DFT calculations were performed using the Vienna *Ab Initio* Simulation Package (VASP).^41^ The outermost electrons of Pb(6s^2^6p^2^), I(5s^2^5p^5^), C(2s^2^2p^2^), N(2s^2^2p^3^) and H(1s^1^) were treated as valence electrons, the electron–ion interaction was modeled with the projector-augmented wave method (PAW).^42^ The electronic interactions were modeled using the PBE+D3(BJ) exchange–correlation functional within the generalized gradient approximation (GGA),^43–45^ and energy and force convergence criteria of 10^−5^ eV and 10^−2^ eV Å^−1^ were used for structure optimization. The calculations were performed with a 4 × 4 × 1 Monkhorst–Pack^46^*k*-grid and a plane wave kinetic energy cutoff of 500 eV.

First, we optimized the C7–C10 structures using the crystal structures provided by Billing *et al.*^47^ These structures were observed at 293 K and belong to the *Pbca* (61) space group. These structures were optimized by only allowing for the positions of the atoms and cell volume to change, but not the cell shape. The optimized C8 and C7 structures were then used to create short-chain structures (C3–C6). For instance, C6 and C5 structures were obtained by removing two carbon atoms from the chains of C8 and C7 structures, respectively, which were subsequently optimized. On the other hand, C4 and C3 structures were obtained by first removing four carbon atoms from the chains of C8 and C7 structures, respectively. Then, the in-plane lattice vectors of C4 and C3 systems were obtained by extrapolating the linear trend of in-plane lattice vectors of the other optimized odd and even systems, respectively; finally, these structures were optimized. The full comparison of geometries of optimized structures with experimental structures is given in Table S1 in the ESI.† Following structure optimization, we performed density of states (DOS) calculations for all our systems using PBE+D3(BJ) exchange–correlation functional within the GGA approach. We employed Gaussian smearing^48^ of width 0.02 eV for partial occupancy of electrons.

## 3 Results and discussions

To investigate a coherent series, films of purely *n* = 1 lead-iodide-based Ruddlesden–Popper 2D perovskites were prepared, containing alkylammonium cations with different lengths of carbon chains. As shown in Table 1, these ranged from ethylammonium (C_2_H_8_N^+^, labeled here as C2) to *n*-decylammonium (C_10_H_24_N^+^, labeled as C10) to form the RP perovskites (C*x*)_2_PbI_4_. For comparison, we also included the methylammonium-based 3D perovskite MAPbI_3_ (*i.e.* C1) in some of the studies.

**Fig. 1:**
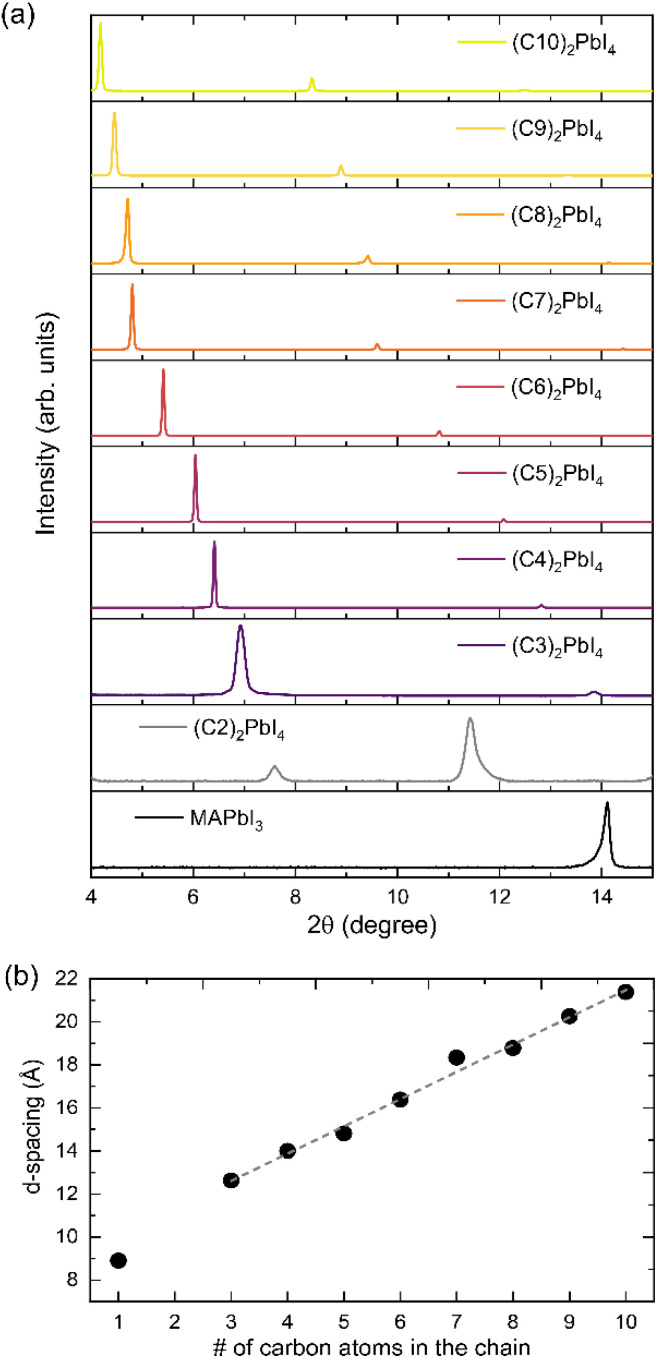
Structural analysis of the perovskite films. (a) XRD patterns of 2D perovskite and MAPbI_3_ with different A-site cations in the range of 2*θ* from 4° to 15°. A wider region is shown in Fig. S5 in the ESI.† (b) Extracted relationship between the number of the carbon atoms in the spacer cation and the resulting *d*-spacing of the perovskite film; note that the C1 sample corresponds to the 3D perovskite MAPbI_3_.

To verify the formation of the 2D perovskite, X-ray diffraction (XRD) experiments have been conducted which are summarized in Fig. 1a, shown in the 2*θ* region between 4° and 15°; wider scans are included in the ESI Fig. S5.† While MAPbI_3_ shows a peak at 14.11°, characteristic of the 3D (110) reflection, all films with larger alkylammonium cations (C2–C10) show peaks well below 10°, indicative of the formation of lower dimensional perovskites. Notably, the diffraction pattern obtained for ethylammonium incorporation, *i.e.* the intended (C2)_2_PbI_4_, is different from the ones with longer alkyl chains and will therefore be discussed separately below.

For the C3 to C10 containing 2D perovskites, pronounced diffraction peaks are observed, corresponding to the (002) lattice plane, which is in good agreement with published results, *e.g.* on C4 and C6.^49,50^ These 2D layers are highly oriented, as commonly observed for these structures, and there are no noticeable impurity peaks (such as lead iodide (001), which would be expected at around 12.6°).^51^ With the increasing length of the organic cation chains, this (002) reflection shifts to lower angles of 2*θ*, as expected from the increased separation of the inorganic sheets. In Fig. 1b, the *d*-spacing of perovskite thin films has been determined from the (002) peak position and is plotted against the number of carbon atoms in the alkyl chain. It can be seen that the *d*-spacing gradually increases from 12.62 Å (for C3) to 21.38 Å (for C10). A fit to the data yields a slope of 1.26 Å, which means that on average each additional C atom increases the spacing by 1.26 Å; note that the reproducibility of the *d*-spacing measurements across different samples is high, with variations being less than 0.1 Å.

To estimate whether the observed lattice spacings influence the coupling between the inorganic sheets and, consequently, the electronic structure of the 2D perovskite, we analyzed the effect of quantum confinement using DFT, as detailed in the ESI Fig. S6.† Notably, we use Cs cations as model systems in these considerations, therefore the calculations do not account for structural effects arising from the incorporation of organic cations but focus solely on the confinement effect. The analysis indicates that the band gap increases with interlayer spacing up to 13.5 Å, beyond which the electronic interaction between inorganic sheets becomes negligible. Comparing this to our XRD measurements in Fig. 1, the transition point at which the inorganic sheets cease to interact is already reached for C4. Therefore, based on this DFT analysis, no further impact by the interlayer distance on the electronic structure is expected beyond C4.

Overall, the XRD results demonstrate the successful incorporation of the C3–C10 alkylammonium cations into highly ordered and phase-pure 2D Ruddlesden–Popper perovskites. This is however not the case for the intended (C2)_2_PbI_4_, as indicated before. As shown in Fig. 1a, two rather broad reflections are seen here, at 7.59° and 11.43°. It is reasonable to assume that the one observed at 7.59° indeed corresponds to the desired (002) reflection of the *n* = 1 2D phase. The one at ∼11.43° has been frequently observed for ethylammonium-based perovskites and is usually assigned to a 1D phase, made of face-sharing octahedra.^35,52,53^ We therefore conclude that ethylammonium is not able to form a phase pure 2D perovskite and it will be excluded from our subsequent study.

Furthermore, we note that for C3 two polymorphs have been reported in the literature. Aside from (C3)_2_PbI_4_ also (C3)_8_Pb_5_I_18_ can form,^35^ which exhibits corner-sharing as well as face-sharing PbI_4_ octahedra. The two polymorphs show reflexes in similar positions in XRD, though the respective (002) reflection of the (C3)_2_PbI_4_ and the (020) reflection of (C3)_8_Pb_5_I_18_ can be distinguished upon close inspection as further discussed in the ESI Fig. S7.† The diffractogram of C3, shown in Fig. 1a, belongs to the (C3)_2_PbI_4_, but depending on the preparation, in particular the film thickness, the unwanted (C3)_8_Pb_5_I_18_ polymorph might form. Throughout this study, the C3 data is included in the discussion, though it should be viewed with caution as we cannot guarantee complete phase purity.

**Fig. 2:**
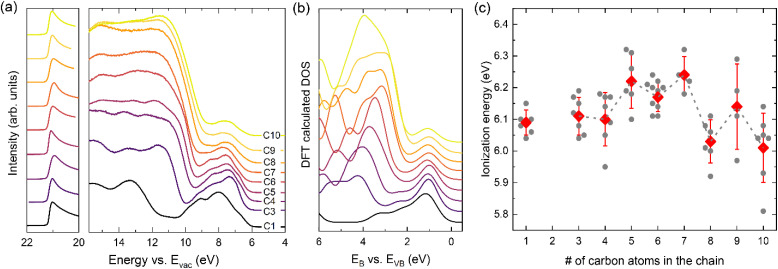
Measurement of the electronic structure. (a) UPS spectra of MAPbI_3_ (C1) and the 2D perovskites (C3–C10) plotted with respect to the vacuum level; the energy of the *x*-axis therefore corresponds to an ionization energy. (b) DFT calculated DOS, showing a good resemblance with the experimental data. (c) Changes in IE with respect to the number of carbons in the alkyl chain. The grey spheres correspond to measurements of different samples, while the red diamond resembles the average value with the standard deviation as an error bar. The dashed line is a guide to the eye, pointing towards variations in IE with carbon chain length.

Since we confirmed the successful formation of 2D thin films for the C3–C10 organic cations, in the following we investigate the effect of spacer length on the electronic and optical properties of the resulting 2D perovskite. To analyze changes in valence and conduction band, UPS and IPES (inverse photoelectron spectroscopy) measurements of the (C*x*)_2_PbI_4_ films were conducted. However, IPES data of sufficient quality could not be obtained for most of these 2D layers. The signal intensity was substantially weaker by a factor of 5–10 compared to that of 3D perovskites, making the determination of a CB onset unreliable. Exemplary data is shown in Fig. S8 in the ESI,† where also the potential causes are discussed; the IPES data is therefore not further considered in this study. The UPS data sets of representative samples of the different (C*x*)-based perovskites are shown in Fig. 2a. While commonly UPS data is plotted with respect to the Fermi level (*E*_F_ = 0 eV), we chose here to use the vacuum level as a common reference (*E*_vac_ = 0 eV). This is achieved by shifting each spectrum along the *x*-axis by the value of its respective work function. As a consequence, there is a common high-energy cutoff (left panel in Fig. 2a) of all data sets at 21.22 eV, which corresponds to the photon energy of the monochromatic UV source. In this type of representation, the value of IE can be directly read out from the onset position of the VB.

By comparing the measured occupied density of states (DOS) in Fig. 2a, trends can be distinguished in the UPS data. Most notably, the intensity for energies above ∼10 eV increases significantly with increasing alkyl chain length, which is mainly due to the contributions of the carbon 2p orbitals. Furthermore, the shape of the front feature, in the spectral region between 6 to 10 eV, changes from exhibiting three distinct contributions to just one single peak. Using DFT, we calculated the electronic density of states of the respective materials. Excellent agreement between the experimental data and the broadened DFT-calculated DOS, shown in Fig. 2b, can be found when assuming that the surface is terminated by the organic cation. This means that with increasing chain length, less and less signal originating from the Pb and I orbitals is observed and the spectrum approaches the one resembling the pure organic cation. A detailed discussion on this trend can be found in the ESI, Fig. S9, S10 and Table S2.† The fact that the organic cation dominates the UPS spectrum calls into question whether a measurement by UPS is able to probe the valence band, which mostly comprises lead and iodide contributions. However, as shown in the ESI Fig. S10,† even in the case of the longest cations studied here, where the relative Pb + I contribution to the overall UPS spectrum is estimated to be only around 15–20% (see Table S2 in the ESI†), the front feature of the VB is still predominantly coming from the inorganic sheet and is therefore representative of the perovskite itself, and not the organic-terminated surface.

From the data shown in Fig. 2a, the ionization energy was extracted and is plotted in Fig. 2c as a function of the number of carbon atoms in the alkyl chain. Here, the VB onset was estimated from the linear extrapolation of the leading VB slope (the procedure is shown in Fig. S11 in the ESI†). Importantly, we analyzed for each of the 2D perovskites several independently prepared samples, indicated by grey spheres. Variations in IE of up to ∼0.3 eV are found for nominally the same material, which shows that for accurate material analysis, it is necessary to perform repeated measurements. The red diamonds indicate the average values, while the error bars correspond to the standard deviation. We suspect that the observed variability of the measured IE for values could have several origins:

Firstly, also for 3D perovskites, it has been reported that even minor variations in the relative ratio of the precursors in the solution can lead to deviations from the intended stoichiometry in the final film, which in turn affect the values of IE and EA, changing it by several 100 meV.^54,55^ Secondly, the measured value of IE could depend on the surface termination. Our previous DFT calculations indicate that a surface terminated by the organic halide will result in an upward shift of the valence band, corresponding to a decrease in IE, compared to a Pb–I terminated one.^56^ However, as discussed in the ESI Fig. S10,† we are confident that for all of the 2D perovskites, the surface is terminated by the organic cation. The same should be the case for MAPbI_3_, where studies by metastable-atom electron spectroscopy find a termination by MA.^57^ Lastly, for 2D perovskites, charging of the sample during measurement occurred due to the lower vertical inter-plane conductivity compared to the 3D materials where such layering is absent. This can been seen for example by a slight shifting of the spectra during subsequent measurements. The effect becomes even clearer when illuminating the samples additionally with white light during a UPS scan, which is known to generate excess charge carriers in a layer that can reduce charging. As shown in Fig. S12 in the ESI,† changes in the width of the spectra, as well as the signal intensity, are observed when comparing measurements under illumination and dark conditions, in particular for the longer spacer cations. Because of this potential charging, the 2D films were kept rather thin (see Materials and methods section) and all measurements were carried out under white light illumination. We believe that the larger error bars for C9 and C10 are caused by inhomogeneous charging, as SEM measurements show significant variations in thickness (see ESI Fig. S4†). When different regions of a sample charge differently, the UPS spectra will be broadened and yield erroneous lower values of IE. Throughout this study, we tried to minimize any possible sources of error in the shown experiments, but we want to raise awareness here that measuring 2D perovskites by photoelectron spectroscopy can be more challenging compared to their 3D counterparts as charging effects can lead to loss of signal and less reliable data.

Coming back to the extracted IE data summarized in Fig. 2c, it can be seen that within the error bars, most of the 2D perovskite films show a similar value of the IE, in the range of 6 to 6.3 eV. This value is similar to the one found for 3D MAPbI_3_ of 6.1 eV. The results therefore indicate that for the material systems studied here, the reduction in dimensionality does not significantly affect the position of the VB. Notably, in this current work, a linear readout procedure is used to extract the VB onset, but sometimes also semi-logarithmic plots are found in the literature. Since this readout procedure affects the extracted values, we included also a log-scale representation of the data in the ESI, Fig. S13.† Notably, also in a semi-logarithmic readout, no significant variations in the IE value are found between 3D and the various 2D perovskites. Here, the values for IE vary between 5.5 and 5.6 eV, when using a dynamic range of three orders of magnitude for the intensity range. As the VB position does not change significantly, the increase in band gap observed in these 2D systems must therefore be due to an upward shift of the CB.

**Fig. 3:**
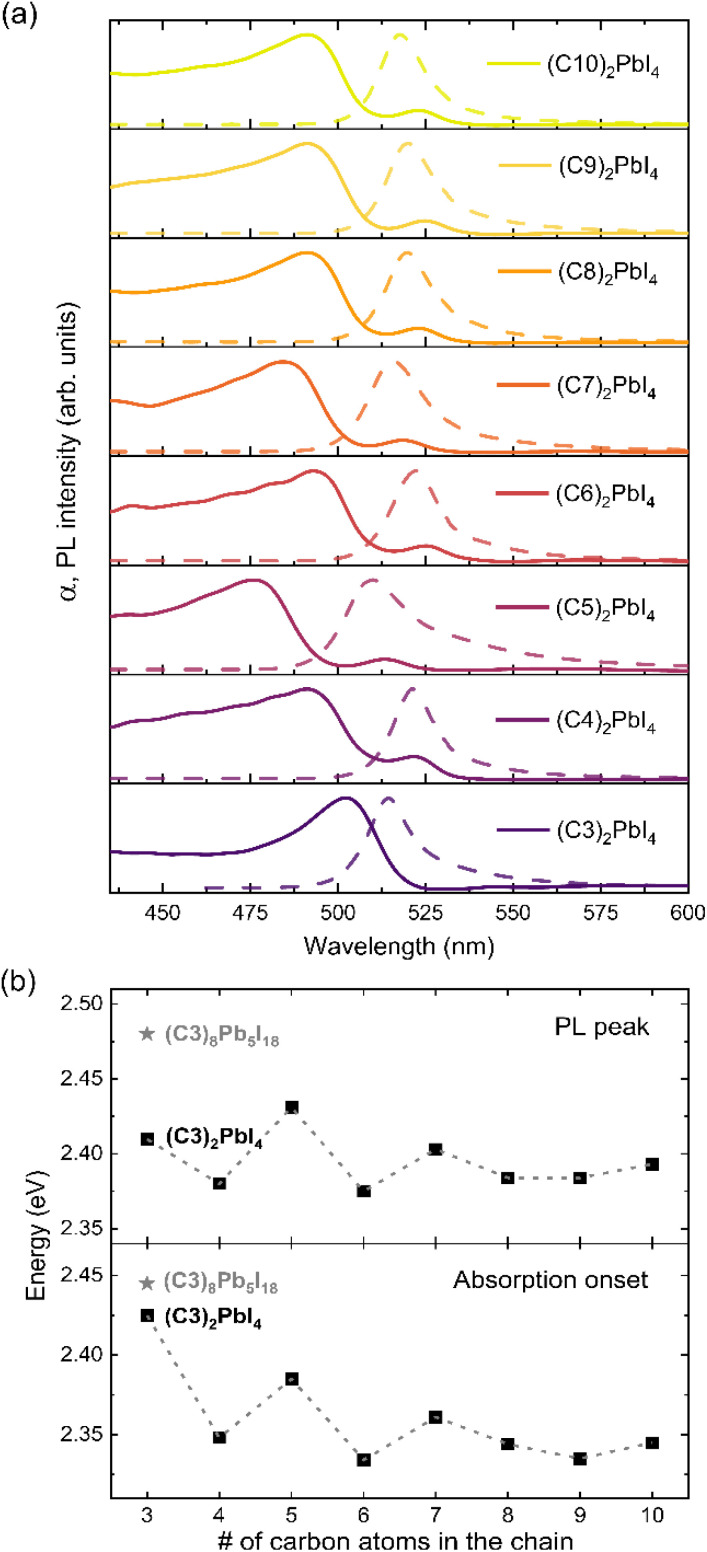
Analysis of the optical properties of the 2D perovskites. (a) The solid line shows the UV-vis absorption which is derived from diffuse reflectance measurements converted by the Kubelka–Munk function for C4–C10 while for the thinner C3 layer reflectance and transmittance spectroscopy were used; a more detailed discussion can be found in the ESI Fig. S14† and the corresponding diffuse reflectance spectra are shown in Fig. S15.† The dashed lines show the steady-state photoluminescence spectra of 2D perovskites. (b) Extracted energy values for UV-vis onset (determination shown in ESI Fig. S17†) and PL maximum *vs.* carbon atoms in the alkyl chain. The second polymorph of C3 is also included in the graph using a star symbol; the corresponding absorption and PL spectra of (C3)_8_Pb_5_I_18_ are depicted in ESI Fig. S16.† The dashed lines act as a guide to the eye, indicating an odd-even effect.

Since measurements of the CB position by IPES were not feasible, we further investigated the changes in band gap energy by optical measurements to complement the UPS data. Fig. 3a shows the absorption coefficient *α* for the different 2D perovskites. Here, the spectra of C4 to C10 were derived using diffuse reflectance measurements following the Kubelka–Munk formalism. Note that here usually the absorption is plotted as *α*/*S*. However, this scattering parameter *S* is commonly assumed to be independent of wavelength^58^ and is therefore omitted here. Using the Kubelka–Munk formalism, instead of the more common transmittance and reflectance experiments, is preferred when highly absorbing materials are investigated, where transmittance tends to saturate over wide regions of the spectra. A more detailed discussion can be found in the ESI Fig. S14† and the corresponding diffuse reflectance spectra are shown in Fig. S15.† Note, for C3 the absorption spectrum has been determined using reflectance and transmittance measurements. Due to the lower thickness of the sample (40 nm) needed to obtain sufficient phase purity and the resulting absence of transmittance saturation the Kubelka Munk formalism is not applicable here.^58^

In Fig. 3a, a double peak feature is observed in the absorption of C4–C10 which has been discussed in the literature and is explained in the framework of free and bound excitons.^59^ Only in the case of C3 a single excitonic feature is present, which we suspect is due to the thinner layers used for C3 (40 nm) compared to the other 2D layers (500–700 nm); a loss of the second feature when going from bulk material to very thin films of 2D alkylammonium halide perovskites has previously been reported.^32^ As outlined above, for (C3)_2_PbI_4_ the use of a significantly thinner perovskite films was required to ensure the formation of the correct polymorph. A comparison of the optical spectroscopy for both C3 polymorphs is presented in Fig. S16 in the ESI.†

A clear excitonic feature is evident in the absorption spectra, attributed to the high exciton binding energy typically found for 2D halide perovskites, which originated from the large dielectric contrast between the lead-halide layer and organic spacers. The absorption onset energy values and the PL peak positions are summarized in Fig. 3b as a function of alkylammonium chain length. Here, the onset of absorption is determined by the respective inflection point (using the second derivative of the absorption spectrum d^2^*α*/d*λ*2, see Fig. S17 in the ESI†), as the Tauc formalism should not be used for excitonic materials;^60^ for C3, the values for both polymorphs (C3)_2_PbI_4_ and (C3)_8_Pb_5_I_18_ are included.

To complement the absorption data, Fig. 3a furthermore contains the photoluminescence (PL) data of the 2D perovskites, measured at room temperature. The absorption onset and PL peak position data summarized in Fig. 3b show that indeed the length of the spacer cation affects the optical properties, *i.e.* the band gap. Surprisingly, there is no linear trend as a function of interlayer distance as expected from a confinement effect, but rather an odd-even trend is observed. Specifically, compounds with an even number of carbon atoms in the spacer (*i.e.* C4, C6, C8 and C10) show only minimal variation in their absorption onset (average of *E*_abs_ for C4, C6, C8 and C10: *E*_abs_ = 2.343 ± 0.006 eV) and PL peak position (*E*_PL_ = 2.383 ± 0.008 eV), which agrees well with an earlier PL study on even-numbered alkyl chains.^25^ In contrast, most of the odd-numbered carbon spacers (C3, C5 and C7) exhibit a blue-shift up to 72 meV in absorption onset and 48 meV in PL peak position; notably, this effect gradually decreases for longer chains, resulting in the observation that C8, C9 and C10 show similar values. Therefore, contrary to the expected trend for a mere confinement effect, which should show an increasing band gap with increasing interlayer spacing followed by a subsequent saturation, here we find that C3, C5 and C7 exhibit the largest gaps; it appears that this widening of the gap is associated with the presence of an odd number of carbon atoms in the spacer cation.

While in organic chemistry, odd-even effects are known *e.g.* for alkene derivatives where alternating changes in packing or melting point are reported,^61^ odd-even dependencies on structural or optoelectronic properties have rarely been reported for halide perovskite systems.^62,63^ The first of these cited studies discusses pronounced changes in charge carrier mobility in tin-based RP perovskites depending on the parity of the spacer cation while in the second reference the authors report that in DJ perovskites the formation of the 2D film is hindered if the odd-numbered diammoniumheptane is used and as a result a 0D structure forms.

**Fig. 4:**
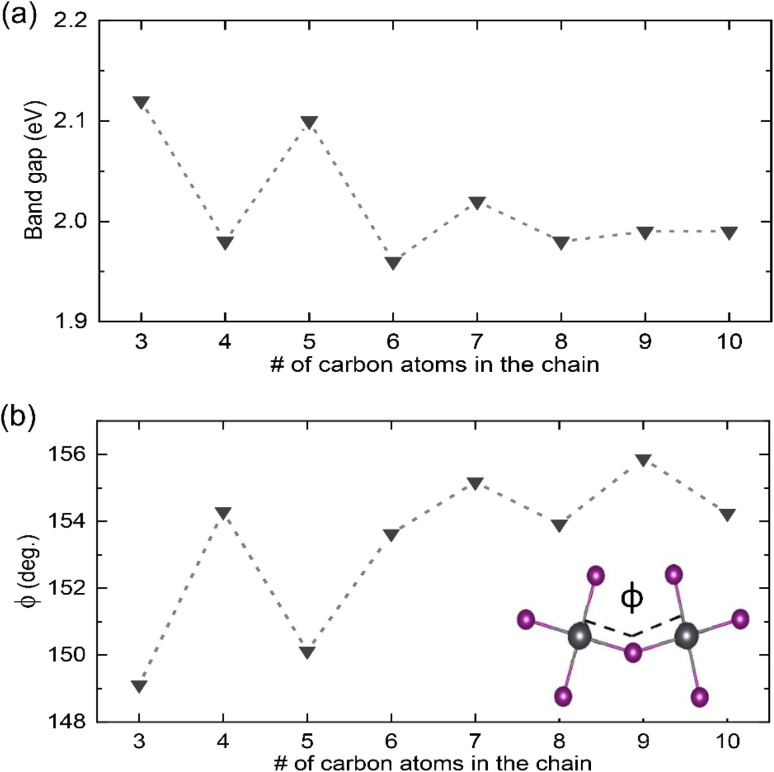
Results obtained from the DFT calculation presented as a function of the number of carbon atoms in the alkyl chain. In (a) the changes in band gaps are shown while (b) summarizes variations in Pb–I–Pb bond angle *ϕ*; the inset in (b) shows the respective bond.

To gain atomistic insight into the underlying mechanism of how the number of carbon atoms in the chain can affect the optical properties of the 2D perovskites, DFT calculations were performed. The DOSs were calculated using the optimized structures of the 2D perovskites (see Table S1 in the ESI†) following the procedure described in the Materials and methods section. The band gaps obtained from these calculations are given in Fig. 4a. In agreement with the trends found in the experimental data, the band gap of the 2D perovskites with even numbered carbon chain is barely impacted by the chain length, with C10 having the highest band gap value of 1.99 eV and C6 having the lowest of 1.96 eV. In contrast, the structures with an odd number of carbon atoms in the chain have a calculated band gap that is significantly larger and then decreases with the number of carbon atoms in the chain, with the band gap of C3 being 2.12 eV and that of C9 being 1.99 eV.

To understand the reason behind these trends, we closely analyzed the optimized structures of these systems. We specifically focused on the correlation between Pb–I–Pb bond angle *ϕ* of neighboring PbI_6_ octahedra and the band gap, which has been suggested as a primary geometrical structure–electronic properties relationship in halide perovskites.^16,64,65^ Here, we observed deviations in the Pb–I–Pb bond angle from its value for the perfect cubic structure (180°) as summarized in Fig. 4b. Indeed, changes in this value follow a similar trend to the changes in band gaps. For structures with even number of carbon atoms in the chain, the angle distortion only slightly differs within the set, ranging from 153.63° (C6) to 154.29° (C4). In contrast, for structures with odd number of carbon atoms in the chain, the angle increases with the number of carbon atoms, with the angle for C3 being 149.11° and that for C9 being 155.88°.

**Fig. 5:**
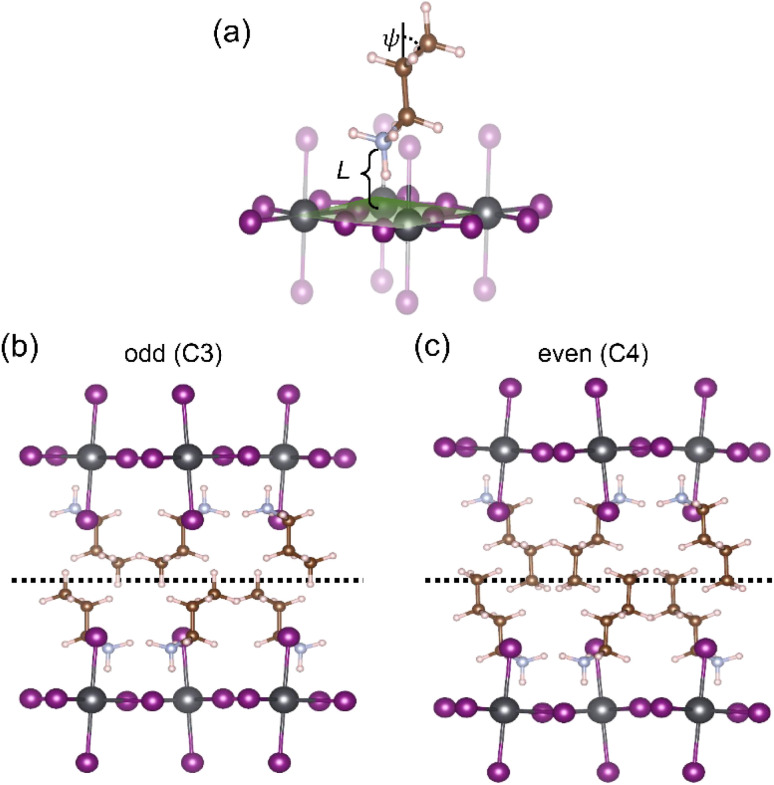
DFT-derived structures of 2D perovskites. (a) Illustration of the vertical distance *L* of the N–PbI_*x*_ plane and the angle *ψ* between the horizontal plane and the tail of the ligands. Examples for packing of the organic ligands within the inorganic lattice for (b) odd and (c) even numbers of carbon atoms in the spacer cation.

We argue the differences observed above for even and odd systems arise from the efficiency of packing/templating of the ligands in the Pb–I framework. This effect is most pronounced for the shortest alkyl chains, where less efficient packing leads to larger distortion. This is evidenced by analysis of the vertical distance (*L*) between the nitrogen atoms and the PbI_*x*_ plane, as indicated in Fig. 5a and b, which is representative of the strength of interaction between NH_3_^+^ and I atoms. As listed in Table 2, this distance is around 2.40 Å for all structures with even number of carbon atoms in the chain. In contrast, for structures with odd number of carbon atoms in the chain, this distance is much larger for C3 (2.63 Å) and C5 (2.55 Å) and continues to decrease to 2.35 Å for C9. Further evidence of the inefficient packing can be seen in the orientation of the final C–C bond, or the tail, of these ligands. As shown exemplarily for C3 in Fig. 5a, in ligands with odd number of carbon atoms, the angle *ψ* of the last C–C bond deviates strongly from the vertical axis, most severely for the shortest alkyl chains with *ψ*(C3) = 57.00° and to a lesser extent for the longer ones *ψ*(C9) = 21.46° (see Table 2 for all values). In stark contrast, for C4 (shown in Fig. 5c) the last C–C bond is nearly vertical at an angle *ψ*(C4) = 2.23°. This results in a distinct difference in packing efficiency for the even numbered chains, such as C4, for which the alkyl chains can interweave as seen in Fig. 5c, compared to the odd ones such as C3 where this arrangement is hindered as visualized in Fig. 5b.

**Table 2:** Vertical distance *L* (along the *z*-axis) between the N atom and the PbI_*x*_ plane as well as the angle *ψ* between the horizontal plane and the tail of the ligands for the optimized structures of all 2D perovskites

	C3	C4	C5	C6	C7	C8	C9	C10
N–PbI_*x*_ plane distance *L* (Å)	2.63	2.47	2.55	2.40	2.40	2.40	2.35	2.38
C–C bond angle *ψ* with horizontal (deg.)	57.00	2.23	39.04	3.8	26.49	12.46	21.46	16.79

Based on the DFT calculations, there is strong evidence that the variations in IE value and band gap originate from the distortions in the inorganic framework, driven by differences in the packing efficiency of the alkyl chains. In such a case, the band gap can be expected to change across structural phase transitions of these 2D materials, which have also been shown to involve changes in the Pb–I–Pb bond angle.^66^ The 2D perovskites under investigation here are known to undergo various structural phase transitions near room temperature, and it has been previously reported that these phase transitions are accompanied by changes in the color appearance of the respective crystals.^47,66^ For example, C5 is monoclinic (*P*2_1_/*a*) at 293 K and it becomes orthorhombic (*Pbca*) slightly above 318 K, while C4 and C6 are already orthorhombic (*Pbca*) at 293 K.^66^ C3 has been reported to be orthorhombic at room temperature^35^ but studies of structural phase transitions are missing.

**Fig. 6:**
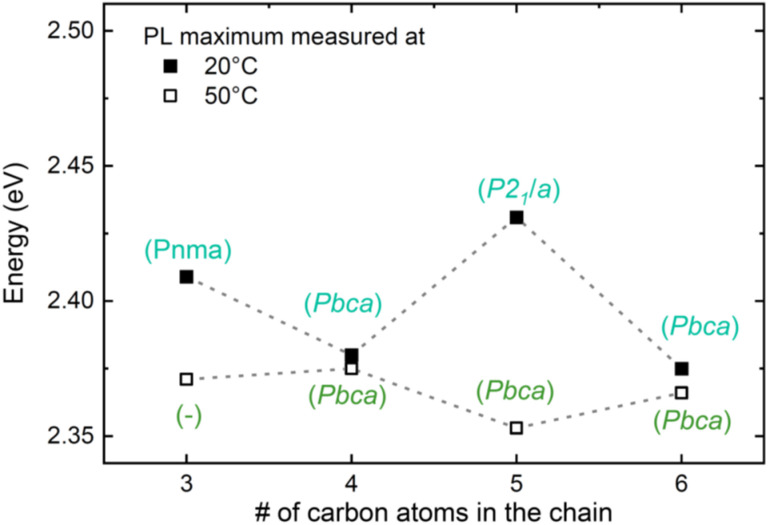
Temperature dependence of the odd-even effect. Comparison of the PL maximum at 20 °C (293 K) and 50 °C (323 K) as a function of the number of carbon atoms in the chain. The referenced crystal structures were assigned based on ref. 35 and 66. The spectra of C3 and C5 are depicted in the ESI Fig. S18a.†

To investigate the impact of structural phases on the optical properties, we also analyzed the PL emission of the compounds C3–C6 at 50 °C (323 K), a temperature at which also C5 has become orthorhombic. The data is shown in Fig. 6, which also contains once more the room temperature (20 °C) PL data from Fig. 3b. Very strikingly, while the PL peak positions of the even compounds C4 and C6 remain almost unaltered upon heating, those of the odd compounds redshift and become similar to those of the even ones. Therefore, the odd-even effect essentially disappears at 50 °C due to the phase transitions, confirming the results found by the DFT calculations that structural distortions are the underlying mechanism. Notably, upon cooling to RT the odd-even effect occurs again for the PL data, as shown in the ESI Fig. S18b;† the transition is therefore reversible.

While the observation of odd-even effects for the electronic and optical properties are in itself already highly intriguing, these findings also raise the question of whether other device-relevant material properties are affected by the distortion induced by the odd-numbered carbon chains. We therefore investigated the effect of the different spacer cations on charge-carrier dynamics and transport in the 2D perovskites. For this, we employed optical-pump–THz-probe (OPTP) spectroscopy. OPTP measures pump-induced fractional changes to the THz electric-field amplitude transmitted through a semiconductor thin film. As previously demonstrated for both bulk and low dimensional lead halide perovskites, OPTP transients measured for thin film samples provide a noncontact measurement of the sheet photoconductivity on ultrafast timescales.^67–70^ Owing to the high degree of orientation of 2D perovskite layers (as seen in Fig. 1a) and to the polarization of the THz probe – both being oriented parallel to the quartz substrate – we can assign the observed photoconductivity signal to the in-plane charge-carrier transport, as previously reported for (PEA)_2_PbI_4_ thin films.^68,70^

**Fig. 7:**
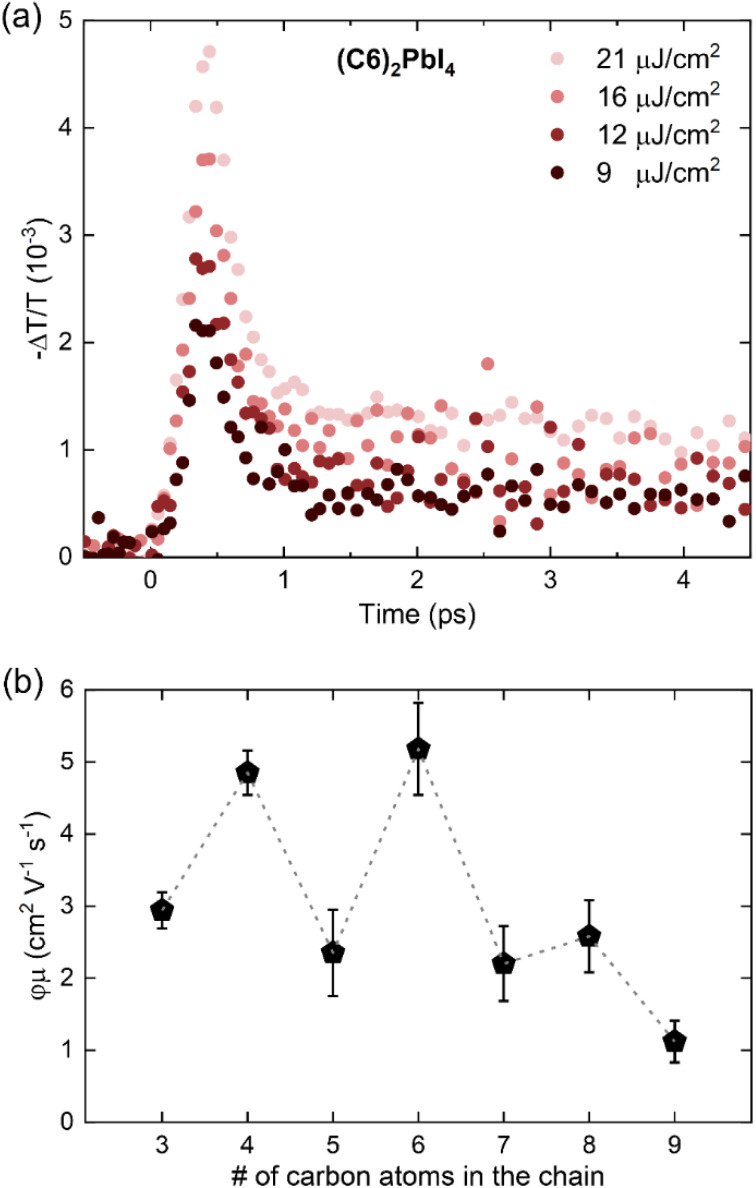
(a) Fluence-dependent OPTP transients for a (C6)_2_PbI_4_ thin film measured following 3.1 eV pulsed excitation with a range of different excitation fluences. (b) Effective THz charge-carrier mobilities for the C3–C9 thin film series plotted as a function of the number of carbon atoms in the cation alkyl chain. The dashed line is a guide to the eye, indicating an odd-even effect in mobility.

As exemplarily shown for a (C6)_2_PbI_4_ film in Fig. 7a, the measured THz photoconductivity decays rapidly, within <1 ps from photoexcitation. Similar ultrafast photoconductivity decays have been reported before for 2D halide perovskites^68,70^ and are observed in this study across the entire C3–C9 spacer series (see Fig. S19 in the ESI†); note that the C10-based perovskite material could not be included in this study, since a sufficiently homogenous sample with respect to layer thickness could not be prepared (see Materials and methods section). While several processes (*e.g.*, trapping, charge-carrier localization, exciton formation)^71–74^ can, in principle, yield such an ultrafast decay of the photoconductivity, Motti *et al.* recently demonstrated that exciton formation processes underlie the observed early charge-carrier dynamics.^68^ Here, the initial THz photoconductivity is dominated by free charge carriers for excitation with photon energies significantly above the band gap, and the fast early decay component can be ascribed to the formation of bound exciton states, following the ultrafast cooling of the highly energetic charge carriers generated by the non-resonant 3.1 eV pump.

Crucially, the ultrafast time resolution of OPTP measurements allows the monitoring of free electron and hole mobilities before fast exciton formation has taken place. It is therefore a useful technique to gain insight into the role of these spacers on charge-carrier transport in the 2D perovskites. We can derive the effective electron–hole sum mobilities *φμ* for the C3–C9 spacer series from the peak THz photoconductivity signal^67,68,75^ as elaborated in the context of Fig. S19 in the ESI.†

The extracted values are summarized in Fig. 7b, where we observe that spacers with an even number of carbon atoms yield significantly higher in-plane charge-carrier mobilities than spacers with an odd number of carbons. While even-numbered spacers – such as C4 and C6 – yield effective in-plane mobilities about *φμ* ∼ 5 cm^2^ V^−1^ s^−1^, which is consistent with previous reports for (PEA)_2_PbI_4_ and (BA)_2_PbI_4_,^68^ odd-numbered spacers – such as C3, C5, and C7 – yield lower effective mobilities in the range *φμ* ∼ 2–3 cm^2^ V^−1^ s^−1^. Furthermore, in spite of the persistence of this odd-even trend, generally lower effective mobilities are observed for C8 and C9 thin films. Crucially, the observed odd-even trend is consistent with trends observed in optical properties and valence band positions across the series and supports previous observations for aromatic spacers in 2D tin perovskites.^63^ Several complementary factors can potentially influence the observed effective mobilities through the C3–C9 series, such as electronic band structure, electron–phonon coupling and excitonic effects.^76^ While the excitonic character does not vary significantly with the spacer chain length, we note that spacer packing-related distortion could significantly influence both band edge dispersion and electron–phonon coupling. Therefore, we posit that Pb–I–Pb distortions – arising from the templating effect of large cations on the [PbI_6_]^4−^ octahedral network^73^ – underlie the observed odd-even mobility effect. It is also worth noting that extrinsic effects such as crystallinity and electronic landscape disorder can, in principle, affect the observed effective mobility.^75,77,78^ While we do not observe significant variations in the crystallinity along the series (see Fig. 1 and XRD discussion), we propose that the observed decrease in effective mobility towards high chain length, *i.e.* for C8 and C9, originates from increased electronic disorder resulting from inefficient packing and coiling of the spacer alkyl chain.

## 4 Conclusion

This study investigated the impact of interlayer spacing on the optoelectronic properties of 2D lead-iodide-based Ruddlesden–Popper (RP) perovskites using alkylammonium cations with varying carbon chain lengths. While phase-pure 2D layers could not be achieved for C2 (ethylammonium), the interlayer spacing could be tuned successfully between 12.62 and 21.38 Å using cations from C3 (*n*-propylammonium) to C10 (*n*-decylammonium). Comparing measurements by UPS with the DFT calculated density of states showed that the surface of all 2D perovskites is terminated by the organic cations. Consequently, the highly surface-sensitive UPS spectra were increasingly dominated by features originating from the spacer cations. Comparing the valence band regions revealed only small variations in the extracted values of IE, which varied between 6 and 6.3 eV using a linear readout procedure for the DOS, with no correlation to the interplane distance. These numbers are surprisingly similar to the value found for 3D MAPbI_3_; note that using a log scale readout these variations were even less and the 3D and various 2D perovskites showed values ranging between 5.5 and 5.6 eV. These results strongly suggest that the valence band position is largely unaffected by reduced dimensionality or interlayer spacing. Consequently, the increase in bandgap observed for 2D *vs.* 3D perovskites must originate from changes in conduction band position, which means that interfaces between these materials are likely not hole-blocking. Due to charging issues caused by the organic cation surface termination, reliable IPES measurements to confirm conduction band shifts were unfeasible.

Despite the small changes in IE, distinct trends were observed, with even-numbered alkyl chains showing mostly lower IE values. This behavior was more evident in optical measurements from diffuse reflectance and PL analysis. Here again, no correlation with interplane separation is found, rather a clear odd-even trend is observed, in which the band gap and emission of all even-numbered carbon chains are identical (*E*_abs_ = 2.343 ± 0.006 eV; *E*_PL_ = 2.383 ± 0.008 eV) while the shorter odd-numbered carbon spacers (C3, C5 and C7) exhibit blue-shifted spectra. This shift diminished with increasing chain length, with C9 matching the even-numbered chains.

DFT calculations confirm that the band gaps have no direct dependence on the interplane distances. This is because the electronic coupling vanishes when the spacer contains more than three carbons in its chain. In contrast, the predicted band gaps successfully reproduce the odd-even trend which can be linked to changes in the Pb–I–Pb bond angle. These differences are driven by the packing efficiency of spacer cations within the Pb–I framework, with packing inefficiency being most pronounced for shorter odd chains.

Finally, the effect of the choice of spacer cation on the electrical properties was tested using terahertz spectroscopy which revealed significantly higher charge-carrier mobility (∼5 cm^2^ V^−1^ s^−1^) within the lead-iodide planes for spacers with even-numbered carbon atoms compared to values for the odd-numbered variety ones (∼2–3 cm^2^ V^−1^ s^−1^).

Our detailed study therefore demonstrates that interplane distance does not play any significant role in the optoelectronic properties. Rather variations arising from the packing of the spacer cations are responsible for observed variations in PL, IE, and in-plane mobility, which in the present study are correlated with an odd-even effect. By highlighting the critical role of spacer cation packing in structural and functional properties, this study provides insights into the rational design of 2D perovskite materials.

## Conflicts of interest

The authors declare no conflict of interest.

## Supplementary Material

TA-013-D5TA01234A-s001

## Data Availability

All data associated with the research presented in this manuscript, including ASCII files of all figures, the DFT-derived partial density of states as well as the CIF files are available at Zenodo at https://doi.org/10.5281/zenodo.15366266.
