# Supplementary material for: Odd-even effects in lead-iodide-based Ruddlesden–Popper 2D perovskites
Source: J Mater Chem A Mater. 2025 May 19;13(24):18935–47. doi: 10.1039/d5ta01234a (PMC12100466; doi:10.1039/d5ta01234a)
Supplement: TA-013-D5TA01234A-s001 [file TA-013-D5TA01234A-s001.pdf]

Supplementary Information (SI) for Journal of Materials Chemistry A.
This journal is © The Royal Society of Chemistry 2025

Supplementary Information

Odd-even effects in lead-iodide based Ruddlesden-Popper 2D perovskites

Maryam Choghaei,[a] Maximilian Schiffer,[b,c] Viren Tyagi,[d] Marcello Righetto,[e] Jiaxing Du, [e] Maximilian Buchmüller,[c,f] Kai Oliver Brinkmann,[b,c] Geert Brocks [d,g] Patrick Görrn,[c,f] Laura M. Herz,[e] Shuxia Tao[d] Thomas Riedl,[b,c] and Selina Olthof ,c[a]*

[a] Department of Chemistry, University of Cologne, Greinstrasse 4-6, 50939 Cologne, Germany

[b] Institute of Electronic Devices, University of Wuppertal, Rainer-Gruenter-Str. 21, 42119 Wuppertal, Germany

[c] Wuppertal Center for Smart Materials & Systems (CM@S), University of Wuppertal, 42119 Wuppertal, Germany

[d] Materials Simulation & Modelling, Department of Applied Physics and Science Education, Eindhoven University of Technology, 5600 MB Eindhoven, Netherlands

[e] Department of Physics, University of Oxford, Clarendon Laboratory, Parks Road, Oxford, OX1 3PU, United Kingdom

[f] Chair of Large Area Optoelectronics, University of Wuppertal, Rainer-Gruenter-Str. 21, 42119 Wuppertal, Germany

[g] Computational Chemical Physics, Faculty of Science and Technology and MESA+ Institute for Nanotechnology, University of Twente, 7500 AE Enschede, Netherlands

[*]Email: solthof@uni-koeln.de

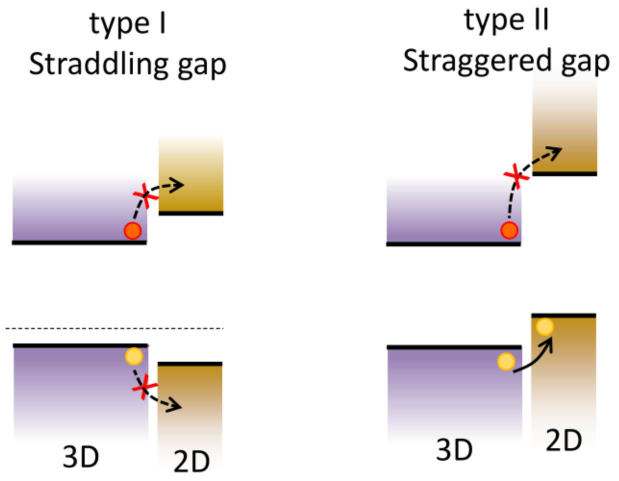

Figure S1: Schematic energy level diagrams for type I (straddling) and type II (staggered) heterojunctions. In the first case, such an interface would inhibit electrons (red ball) as well as holes (yellow ball) from entering into the wide gap semiconductor (the 2D perovskite in this example). In the case of the staggered junction depicted here, hole transfer would be efficient, while electrons are blocked.

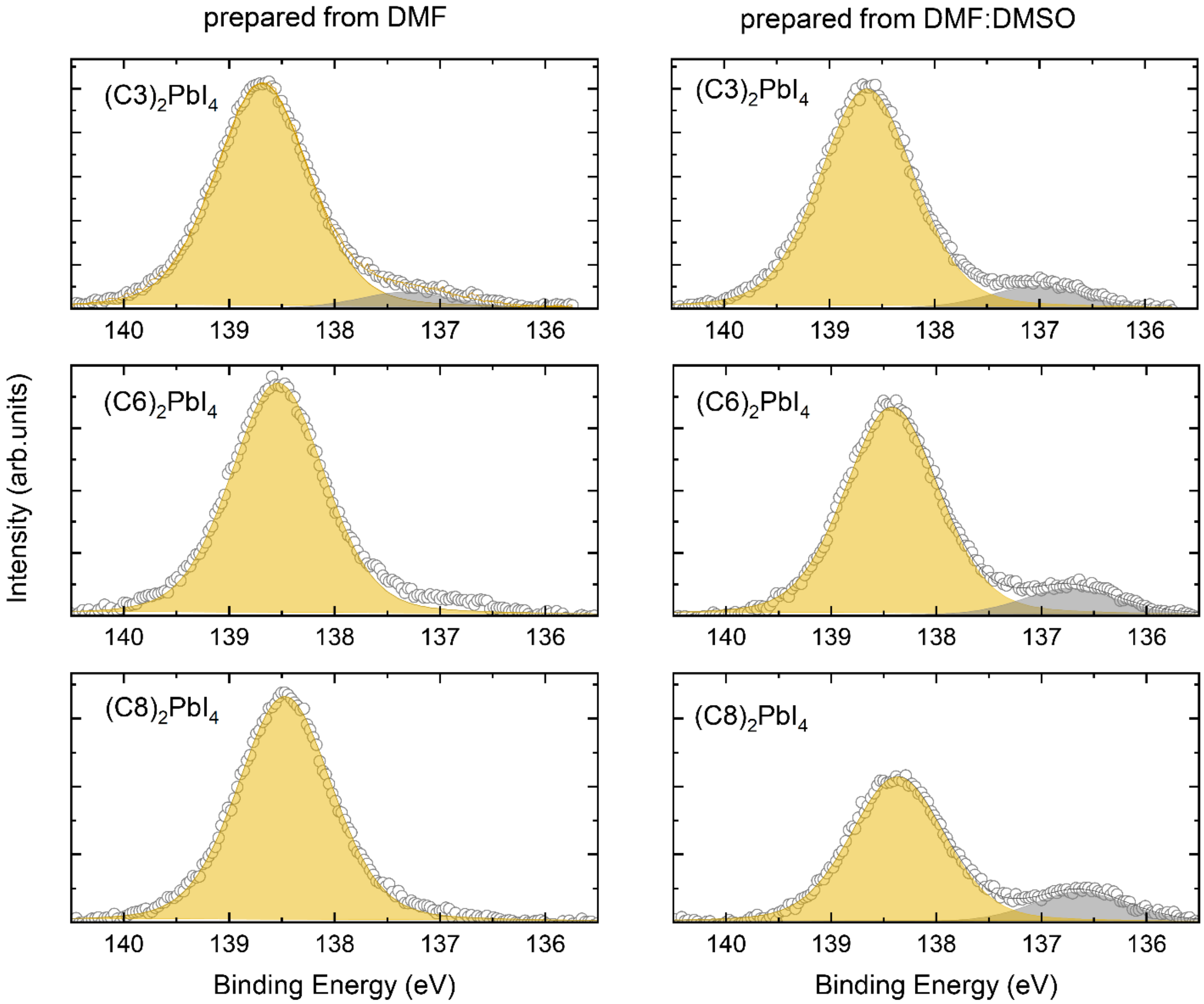

Figure S2: Exemplary XPS measurements of the $Pb4f_{7/2}$ peak for 2D perovskites show the solvent-dependent formation of metallic lead. Samples shown on the left were prepared from DMF, while for the ones on the right a mixture of DMF and DMSO was used (4 : 1). The data reveals that a significant amount of metallic lead is formed when DMSO is involved in the preparation, which is why all samples were prepared using exclusively DMF throughout this study. XPS measurements were done using the same hemispherical electron analyzer as for UPS measurements, while a non-monochromated Mg Kα x-ray source (VG) was used for excitation.

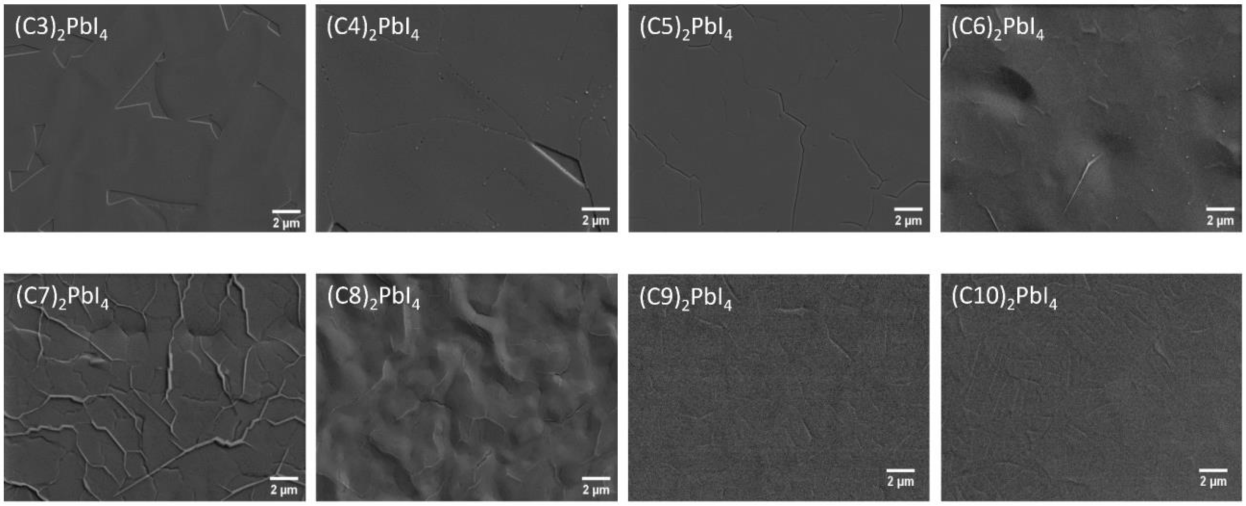

Figure S3: Top-view scanning electron microscopy (SEM) images of 2D perovskite thin films with varying spacer chain lengths (C3–C10) deposited on different substrates. Films based on shorter alkyl chain spacers (C3 and C4) were deposited on PEDOT:PSS-coated substrates. In contrast, films with longer chain spacers (C5 through C10) were fabricated on bare ITO substrates. SEM imaging was carried out using a Zeiss Neon40 setup at an acceleration voltage of 5 kV and an SE2 detector. Scale bars are included for reference in each image.

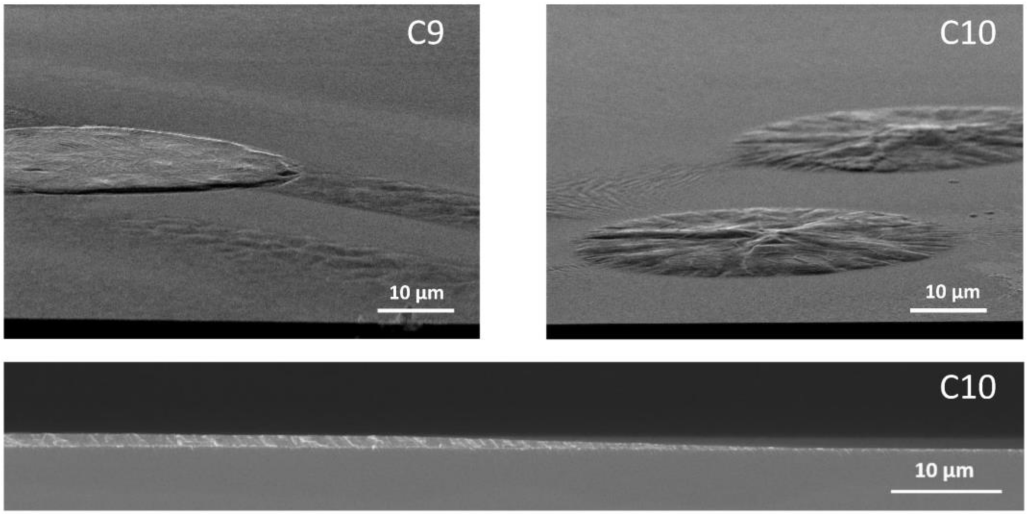

Figure S4: SEM images showing the non-uniform film formation of the perovskites with the longer-chained cations. The top images show a 15° tilted view of (C9)$_2$PbI$_4$ and (C10)$_2$PbI$_4$ revealing large-scale structures, measured with a Philips XL 30S at 10 kV acceleration voltage and an InLense detector. The bottom image is a cross-sectional SEM image of the C10-based perovskite showing variations in thickness from 100 to 1000 nm. This measurement was taken using a Zeiss Neon40 setup at an acceleration voltage of 5 kV and an InLens detector.

This uneven film distribution can affect in particular the UPS measurement due to partial charging of the samples, which can lead to broadening and therefore errors in the extracted energy values.

Table S1. Lattice parameters of all the optimized DFT structures which are orthorhombic and belong to the Pbca space group. These are compared to experimentally determined structures obtained from Reference 1 for C7 – C10 and from Reference 2 for C4 – C6.

Label	Type	a (Å)	b (Å)	c (Å)
C3	DFT	8.81	8.52	24.88
C4	Exp.	8.87	8.69	27.60
	DFT	8.95	8.67	26.72
C5	Exp.	9.00	8.73	29.95
	DFT	8.85	8.55	29.66
C6	Exp.	8.94	8.68	32.70
	DFT	8.94	8.65	31.70
C7	Exp.	9.01	8.70	34.56
	DFT	8.93	8.63	34.25
C8	Exp.	8.98	8.68	37.48
	DFT	8.89	8.60	37.12
C9	Exp.	9.02	8.70	39.75
	DFT	8.94	8.62	39.40
C10	Exp.	8.98	8.67	42.52
	DFT	8.92	8.62	42.27

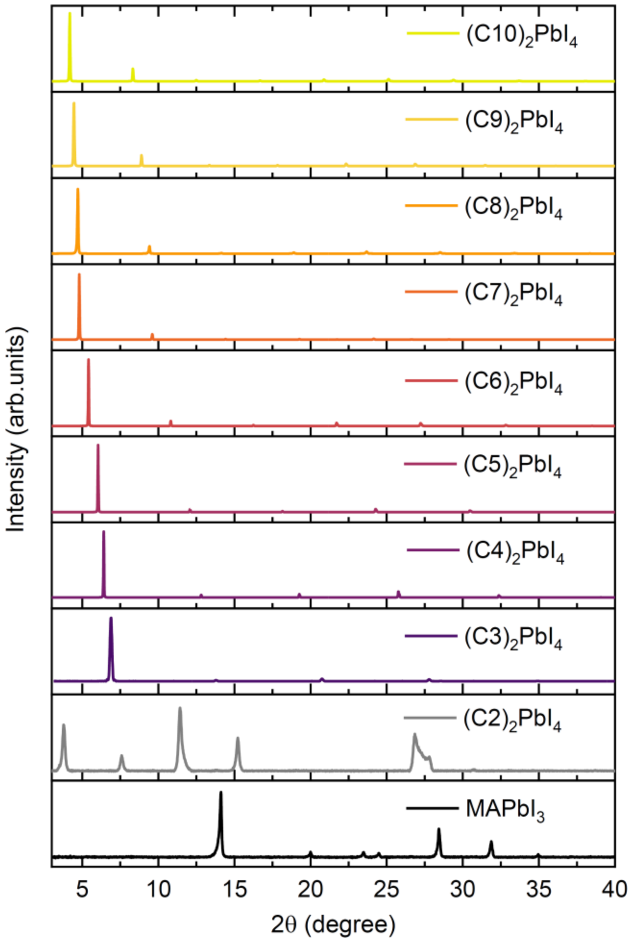

Figure S5. XRD patterns of $MAPbI_3$ and the 2D perovskite with different alkyl spacer cations. This is the same data set as Figure 1a of the main article but shows a wider 2θ region.

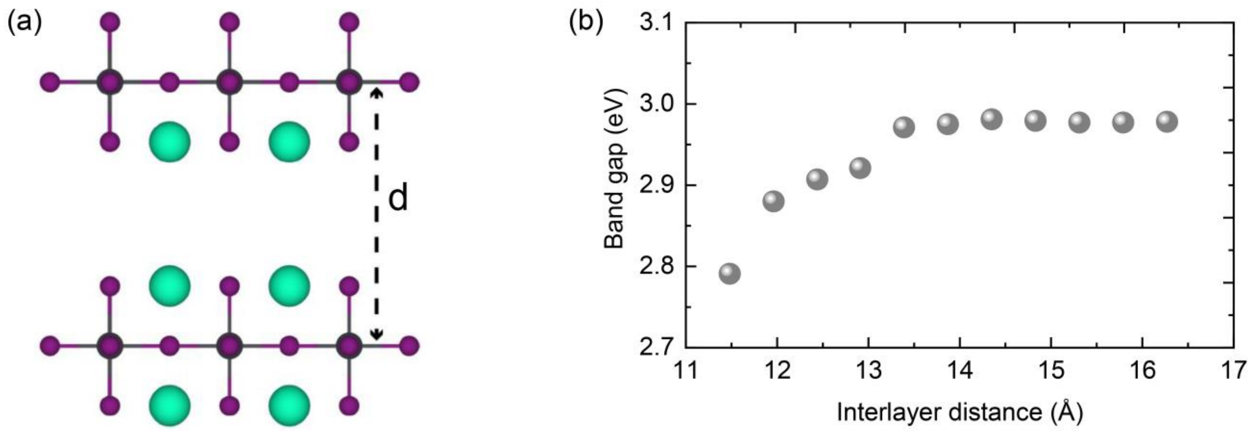

Figure S6. Quantum confinement effect: We study the possible quantum confinement effect of these 2D perovskites by increasing the distances between Pb-I inorganic layers. To avoid the impact of the organic spacer cation on the band gap, we used as model systems Cs^+ as the cation between the inorganic layers and varied the separation between the inorganic layers as shown in (a). We calculated the DOS of these model systems by treating the outermost electrons of Pb($6s^2 6p^2$), Cs($5s^2 5p^6 6s^1$), and I($5s^2 5p^5$) as valance electrons, the electron-ion interaction was modeled with the projector-augmented wave method (PAW). The electronic interactions were modeled using the PBE+D3(BJ) exchange-correlation functional within the generalized gradient approximation (GGA), and energy and force convergence criteria of 10^{-5} eV and 10^{-2} eV/Å were used for structure optimization. The calculations were performed with a 4×4×1 Monkhorst-Pack k-grid and a plane wave kinetic energy cutoff of 300 eV, and we employed Gaussian smearing of width 0.02 eV for partial occupancy of electrons.

The band gaps at different interlayer spacing obtained from these calculations are given in (b). As evident from the Figure, the band gap increases with interlayer spacing until 13.5 Å, after which increasing the interlayer spacing hardly impacts the band gap. From these results, it can be deduced that the quantum confinement effect influences the band gap of these 2D perovskites studied here up to an interlayer spacing is equivalent to that of the C4 structure.

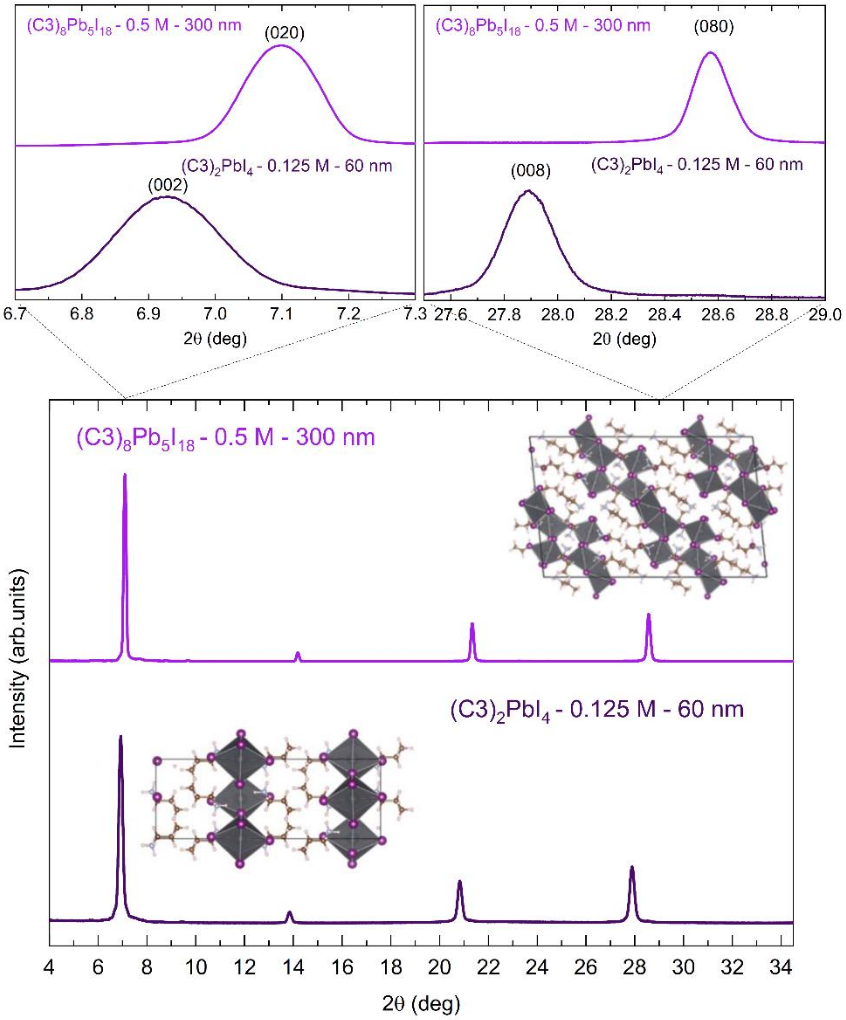

Figure S7: XRD of C3-containing films prepared from either 0.5 M or 0.125 M precursor solution, showing that the different layer thickness result in two distinct polymorphs $(C3)_8Pb_5I_{18}$ and $(C3)_2PbI_4$; the polymorphs can be distinguished by the peak shift. The corresponding crystal structures are included in the respective diffraction pattern and show corner-sharing octahedra in $(C3)_2PbI_4$ and additional face-sharing octahedra in $(C3)_8Pb_5I_{18}$. A detailed view of two distinct reflexes is depicted in the upper panel, visualizing the diffraction shift. A different diffractometer was used for these measurements compared to the measurements included in the main article, namely a PANalytical X'Pert PRO.

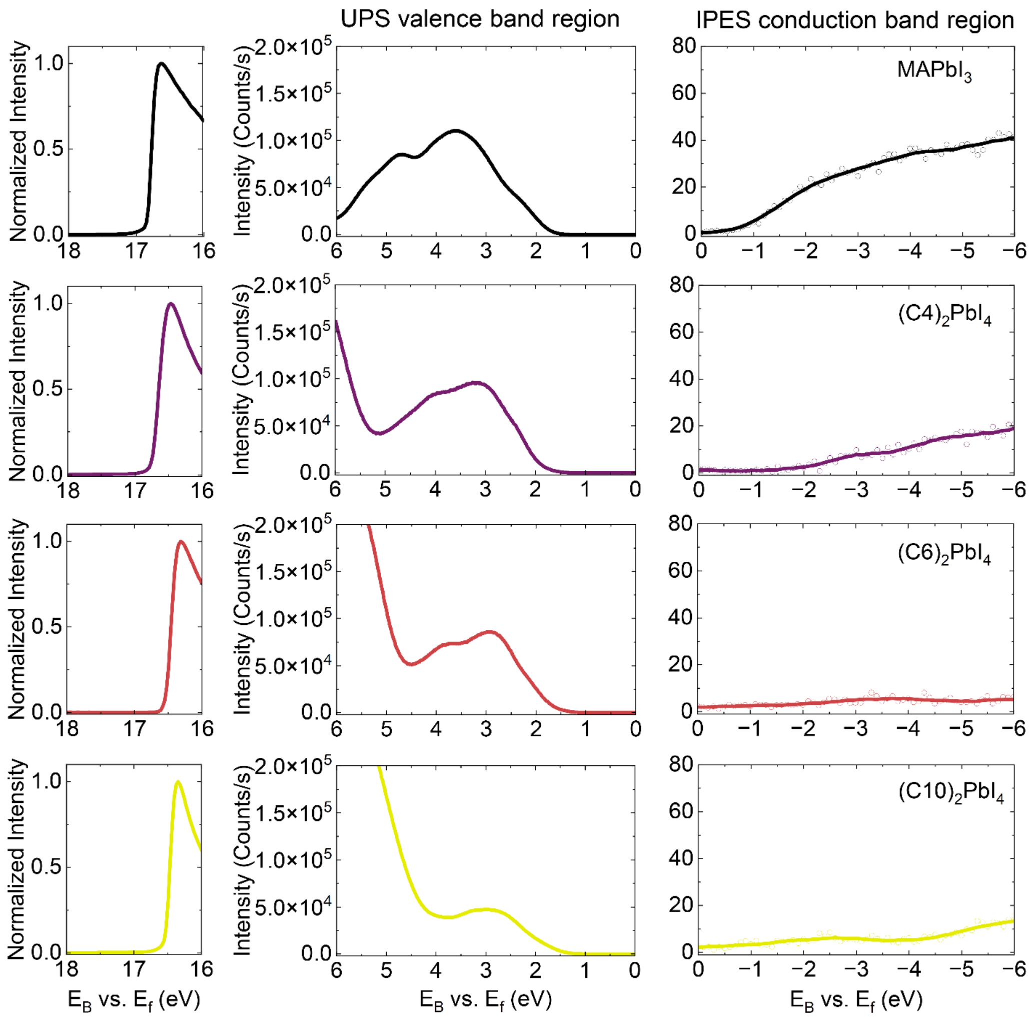

Figure S8: Selected examples of UPS and IPES spectra for 3D and 2D perovskite samples, as indicated in the graph, this time plotted with respect to the Fermi level E_f. Note that the intensity scale of the VB region (center panels) and CB region (right panels) are kept the same to visualize variations in signal intensity with increasing spacer cation length. IPES measurements were done using a Kimball Physics electron source (ELG-2) for excitation and detecting the emitted photons via an SrF_2/NaCl bandpass detector (IPES 2000, Omnivac).

For both UPS and IPES, a clear lowering of the signal with increasing C*x* is seen. As discussed in the main article, as well as Figures S9 and S10 below, the intensity drop in UPS data in the binding energy region of ~ 2 to 5 eV can be attributed to the surface termination by the organic cations. For this highly surface-sensitive technique, this termination suppresses the DOS here which mostly originates from the inorganic sheets. While for UPS measurements, the data remains sufficiently intense for data evaluation, the same is not the case for the IPES data. Here, beyond (C4) the determination of the onset becomes highly unreliable. In addition to the loss in signal intensity, also potential charging and degradation of the surface by the electron beam are possible, which is why it was decided not to include the IPES data in the overall analysis of this study.

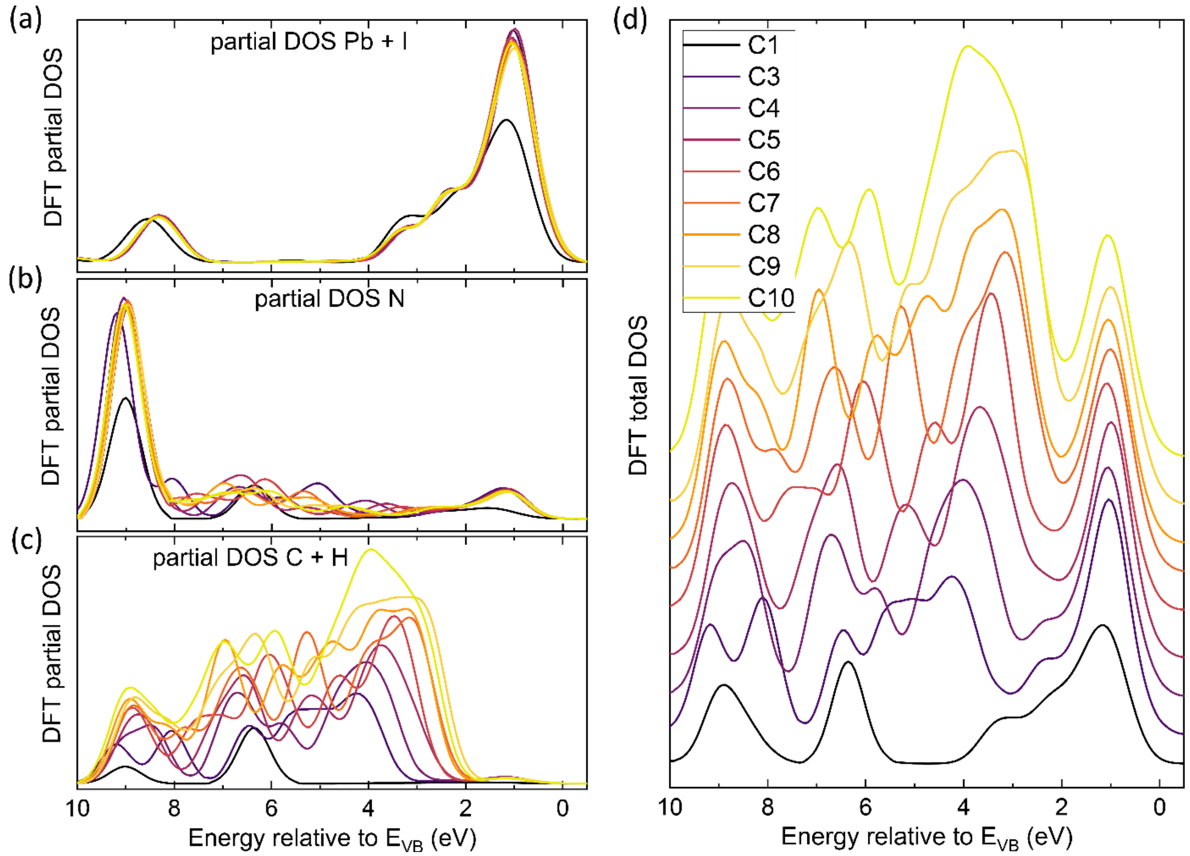

Figure S9: DFT calculated partial DOS for the 2D perovskite with the different C*x* cations, shown only for the occupied DOS; the reference 0 eV is set to the VB onset. The DFT data is broadened by a Gaussian function as previously described in the Supplementary Material of Reference 3 to match the experimental resolution of the UPS measurement. Furthermore, the energy axis of the calculated data was stretched by a factor if 1.14 (for the Pb and I contribution) and 1.3 (for C, H, N contributions) which was motivated by a thorough comparison to the experimental UPS data. Sub-Figure a) shows the partial DOS of the inorganic framework originating from the Pb and I atoms. While slight differences are found between the 3D perovskite (C1, i.e. $MAPbI_3$) and the 2D materials, no variation in the partial DOS is found with the length of the organic cation. b) shows the partial DOS of the nitrogen atoms. Here, also a slight difference is seen between the 3D perovskite, where the contribution to the front VB feature (0 to ~ 2eV) is negligible for the 3D case, but more pronounced for the 2D. The strongest differences in DOS for the different materials are observed for the C and H contributions to the DOS which are summarized in c). While states corresponding to MA^+ are only found at binding energies higher than ~6 eV, the alkyl chains also contribute significantly in the region between 2 and 6 eV. Also here, small contributions are found in the front VB region (< 2eV) in the case of the 2D perovskites. Finally, d) shows the combined total DOS, achieved by adding up the data shown in a)-c); here the curves are vertically shifted to enhance clarity. Since the main contribution at low binding energies is originating from the Pb and I atoms, which are not changed by the different spacer cations, the front feature of the VB is not significantly affected by the choice of C*x*. As discussed in the main article, this is however not what is experimentally observed, therefore the probing depth of UPS has to be considered, as described next in Figure S10.

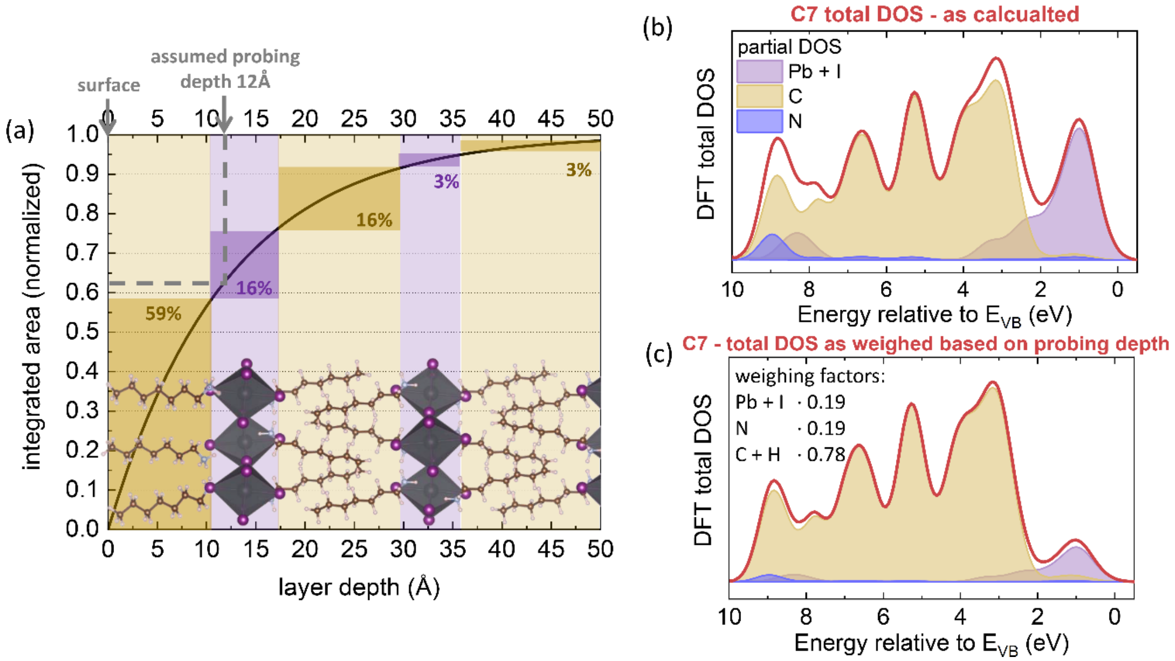

Figure S10: Visualization of the estimation of the probing depth λ to estimate how a surface terminated by the long alkyl chains should affect the overall appearance of the UPS spectrum. The curve shown in a) was created by (i) first assuming a probing depth *λ* of UPS of 12 Å (based on extensive experience with organic bilayers, e.g. Reference 4 and using the equation $d=\lambda \cdot ln(I_0/I)$ for the relation between layer thickness *d* and UPS signal intensity *I*. (ii) Next, this equation was integrated, as the area under the curve corresponds to the overall UPS signal measured for a sample. (iii) Finally, for simplifying the readout, this integrated function was furthermore normalized – resulting in the curve shown in this image. In this plot, the probing depth λ can easily be discerned gain, since per definition it's the layer thickness after which the signal drops to 1/e ≈ 0.37. Therefore, it is located at 0.63 in this plot and the initially set probing depth used in the calculation of 12 Å can directly be read out again (as indicated by dashed line). The crystal structure inserted in the graph visualizes how much of the signal in a UPS measurement is expected to originates from the different regions of the 2D perovskite crystal in the case of C7. While here the surface terminating alkyl chains constitute 59% of the signal, the first inorganic sheet is expected to merely contribute ~16%. For the deeper lying layers, the remaining signal further decreases exponentially. Summing up over the different layers, it can be expected that ~ 78% of the signal measured by UPS will originate from alkyl chain contributions (C + H) indicated by dark yellow color, while the Pb + I + N contributions situated in the purple regions make up ~ 19%. Therefore, the UPS measured DOS will not resemble the DFT calculated bulk DOS, but rather the different contributions have to be weighed. This is compared in sub-Figures b) and c), where the DFT DOS is compared for the as-calculated DOS (same data as shown in Figure S9d), and below that is the case where the individual partial DOS was weighed by the factors extracted from the probing depth consideration. A much better agreement between theory and UPS experiment if found for the latter case, which is a strong indication that indeed for all of the samples the surface is terminated by the organic cation, which contributes increasingly more to the overall DOS the longer the cation is. Table S2 summarized the weighing factors for all samples.

Table S2. Calculated contributions of the organic and inorganic layers of the perovskite 2D surface to the overall signal when performing a surface sensitive UPS measurement. As discussed in Figure S10, due to the low probing depth, the surface termination can significantly alter the contributions seen from the different film constituents. The values listed here were used to produce the weighed total DOS DFT plots shown in Figure 2b of the main article.

	C1	C3	C4	C5	C6	C7	C8	C9	C10
Pb + I + N contribution (%)	56	36	30	27	21	19	18	17	14
C + H contribution (%)	42	62	69	71	75	78	81	83	85

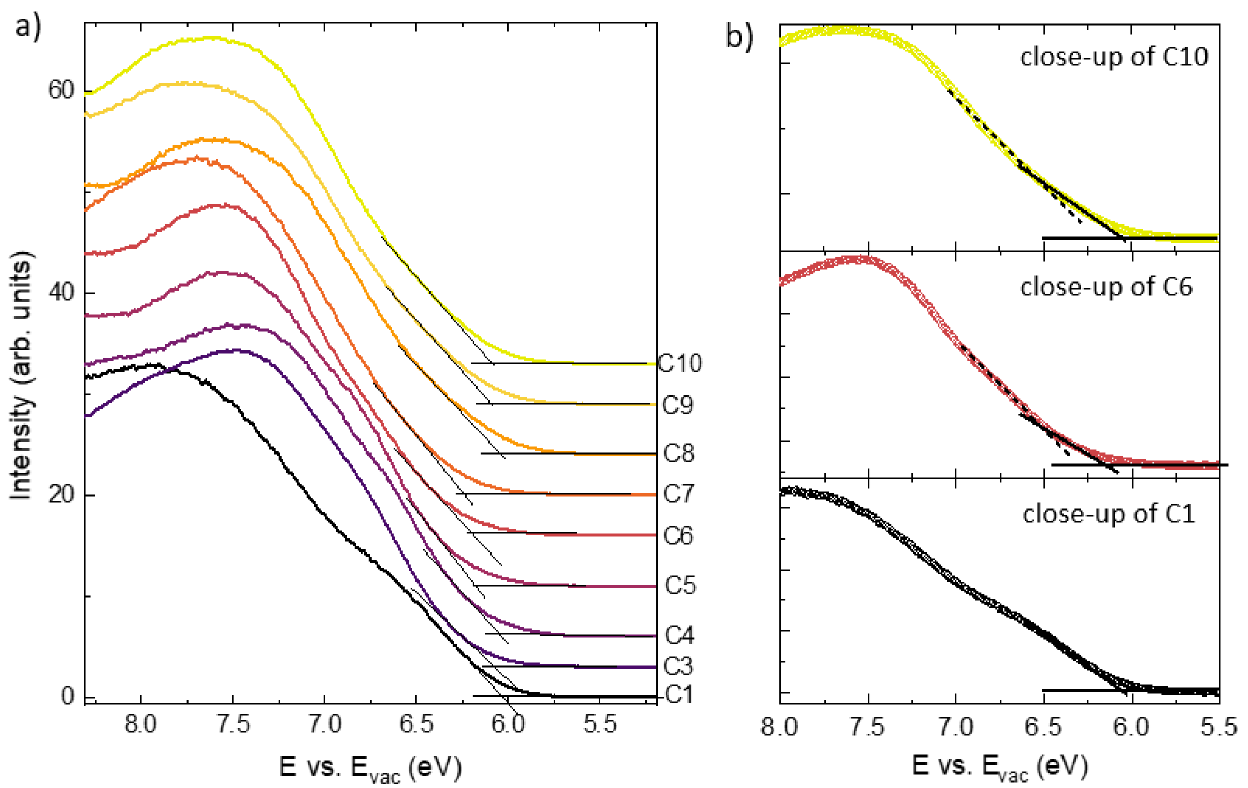

Figure S11: Close-up of the front VB feature of the UPS data of representative samples. Note that here the height of the VB features is arbitrary, since in contrast to the data shown in Figure 2a) of the main article, here the spectra have been multiplied to achieve a similar intensity of the VB feature, which helps to visualize differences and similarities better. In a) the measurements of the 3D and 2D perovskites are shown including the linear readout procedure used in this study. While for the 3D perovskite (C1) one clear leading edge is seen, all the 2D layer exhibit two slopes. This is better visible in sub-figure b) where for the examples of C10 and C6 the two separate slopes are indicated. It should be noted that for the same material there is some sample-to-sample variation in the strength of the front feature, in the examples shown here for C10 it is more pronounced than for C6. This variation can make it challenging to consistently evaluate the onset and care has to be taken to analyze the different samples within a series as well as the different C*x* materials consistently.

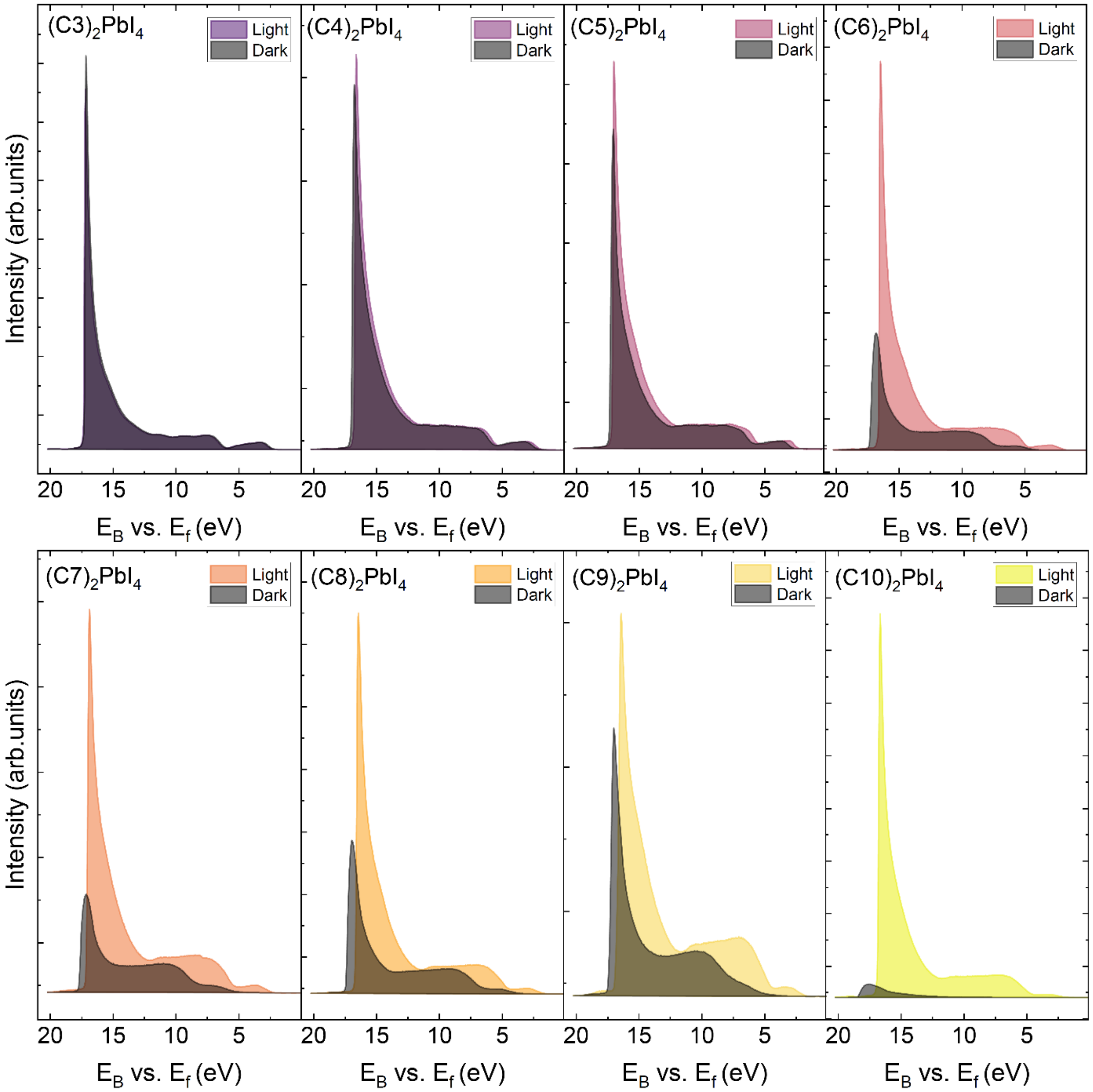

Figure S12: UPS data of the 2D perovskites showing the effect of charging during measurement due to insufficient conductivity. In contrast to the main article, the x-axis is plotted as measured, therefore relative to the Fermi energy E_f. Each panel contains two scans, one conducted in the dark and the other under white light illumination. Notable differences already appear for C5 and become increasingly more pronounced for the larger organic cations. The shifting and loss of signal intensity for scans in the dark indicate an insufficient conductivity of the samples. Here, the removal of the photo-electrons leads to the accumulation of holes at the surface which charge up the sample and compress the overall width of the spectrum (thereby affecting the extracted IE value).

The white light illumination is known to alleviate this issue to some degree by generating excess charge carriers in the bulk which can reduce charging. Due to this issue such 2D films have to be kept thin and in this study all measurements were carried out under white light illumination.

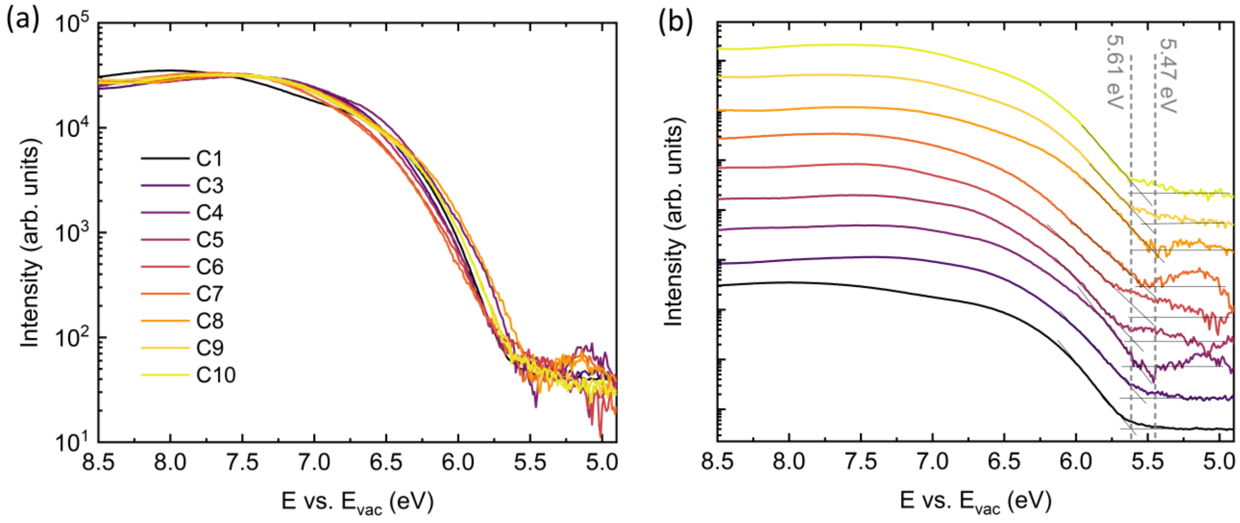

Figure S13: To investigate how DOS drops in band gap, we also include the VB UPS data in a log scale representation. In a) all data sets are set to the same dynamic range, by adjusting background and VB peak intensity. It is important to note, that in a log-scale the chosen (or setup-given) dynamic range affects the readout of the onset of the DOS (see e.g. Supporting Information of Reference 5); here we aimed for 3 orders of magnitude, which is most commonly displayed in publications. In this graph it can be seen that the onset of the DOS is very similar for 2D and 3D perovskites, with no indication of a difference in steepness. In b) the same data is plotted but vertically shifted (by multiplication), to make the individual scans better visible. The so-determined VB onset in the log scale only changes by ~ 100 meV for this data set, so in agreement to the conclusions drawn from the linear readout, also here we find that the change in dimensionality (3D→2D) or increase in spacer length (C3→C10) barely affects the ionization energy.

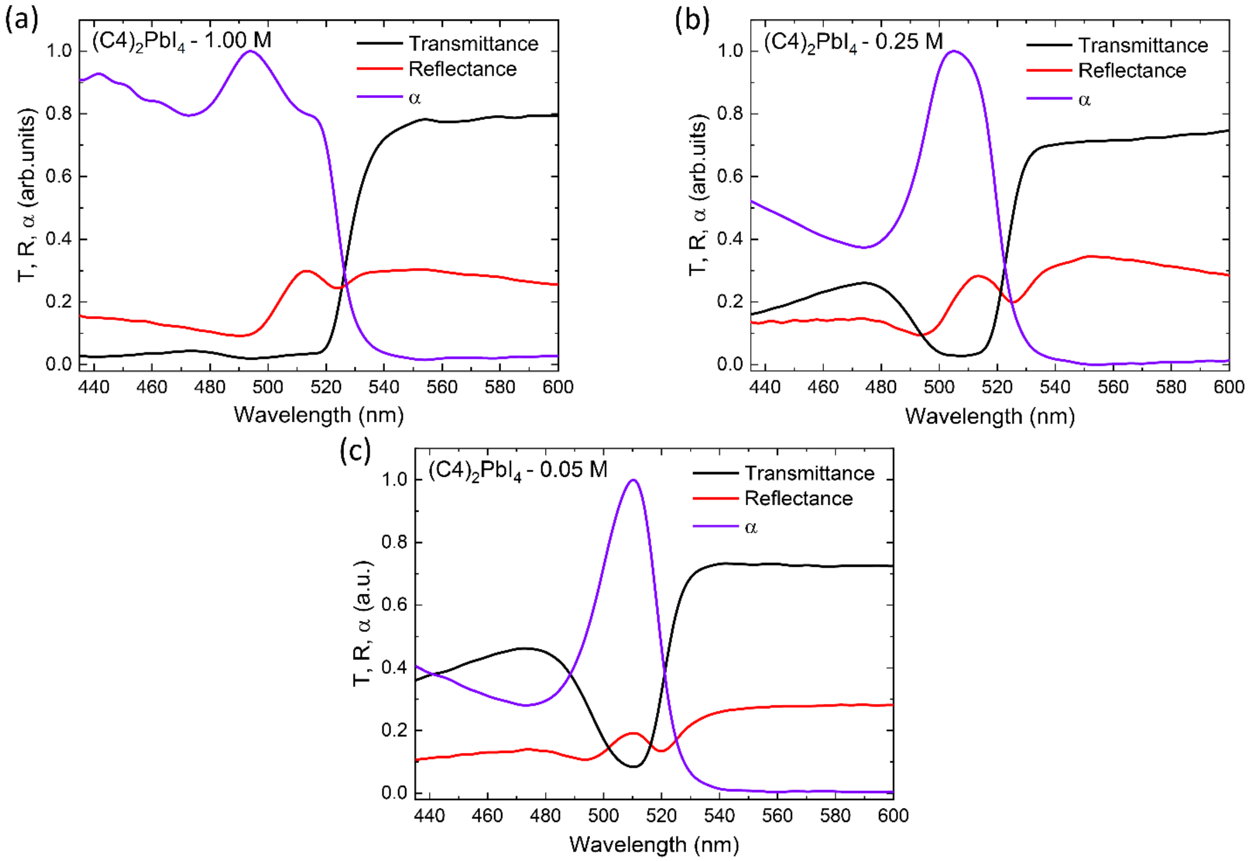

Figure S14. This Figure is intended to motivate the choice of the Kubelka-Munk method to evaluate the absorption of the highly absorbing 2D perovskites. This method assures that the absorption is accurately measured. The sub-figures show spectra of transmittance (T), reflectance (R), and absorption coefficient (α) using films prepared from (a) 1 M, (b) 0.25 M, and (c) 0.05 M precursor solution of C4 samples. For samples exhibiting strong spectral dependence in diffuse and specular reflectance, absorption is commonly assessed by diffuse reflectance (R_{diff}) or measuring the reflectance (R) and transmittance (T) in an integrating sphere. The latter approach derives the absorption coefficient $\alpha \propto -\log\left(\frac{T}{1-R}\right)$, which is not suitable in case of most single crystals or highly absorbing samples where T saturates within the dynamic range of the spectrometer, consequently leading to an underestimation of α in the affected spectral range.

The spectra illustrate transmittance saturation in (a) and (b) over a broad spectral range, indicating that lower concentrations are necessary for accurately measuring the absorption of the present 2D Ruddlesden-Popper series. Furthermore, we observed that the wetting for C7 - C10 is poor for lower concentrations, prompting the use of an alternative measurement technique within the present setup. By measuring the diffuse reflectance R_{diff}, we derive the absorption coefficient α using the relation $\alpha/S = \frac{(1-R_{diff})^2}{2R_{diff}}$, assuming a constant arbitrary scattering parameter *S* over the observed spectral range. As the value of S is considered to be independent of the wavelength (Reference 6), *S* is omitted in Figure 3 of the main article. Notably, the diffuse reflectance relation is valid assuming an infinitely thick opaque layer (Ref. 6). Therefore, the technique is not applicable to measure $(C3)_2PbI_4$, in this study, which could only be prepared with sufficient phase purity at thin layers of ~ 40 nm.

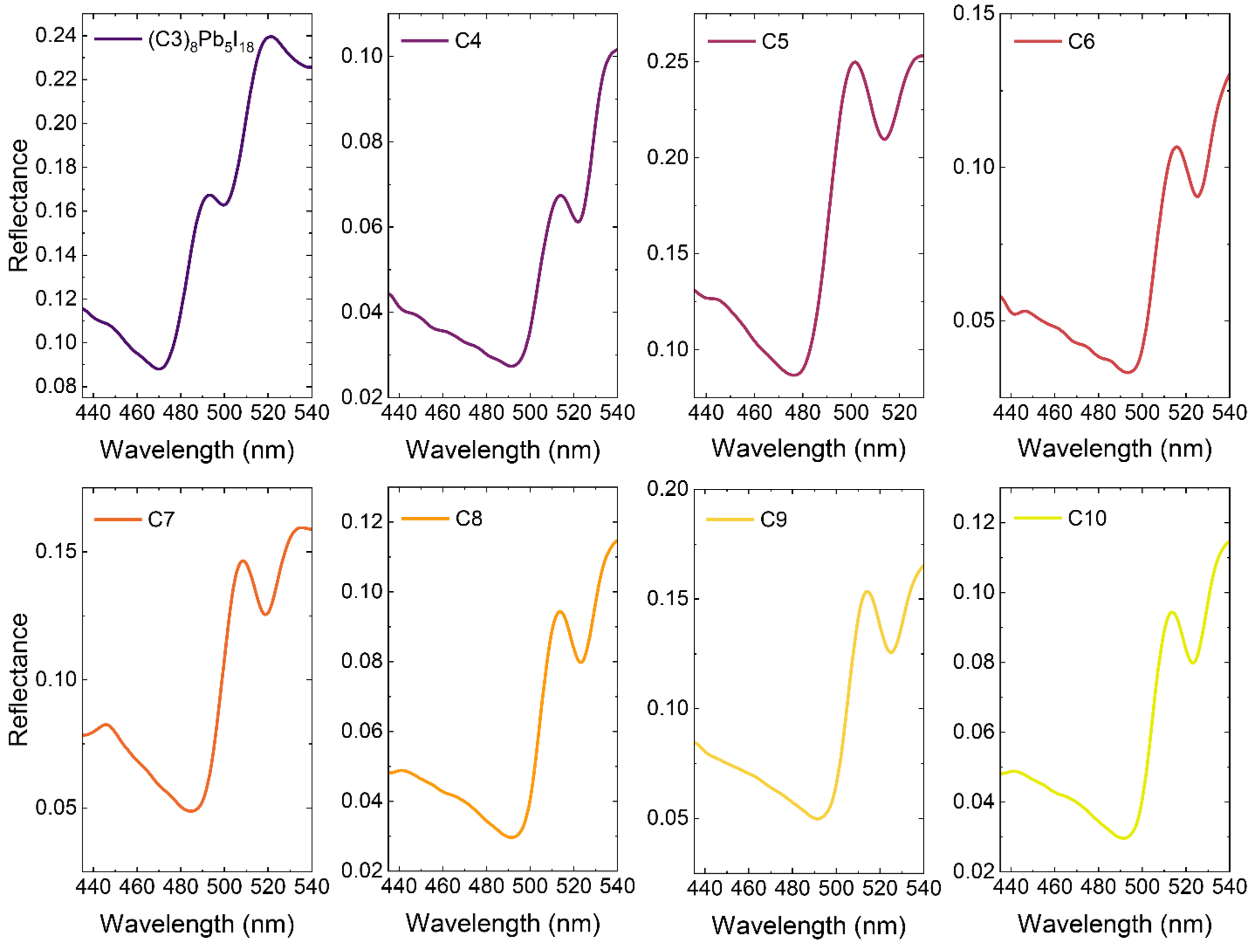

Figure S15. Diffuse reflectance spectra of 2D perovskites and $(C3)_8Pb_5I_{18}$.

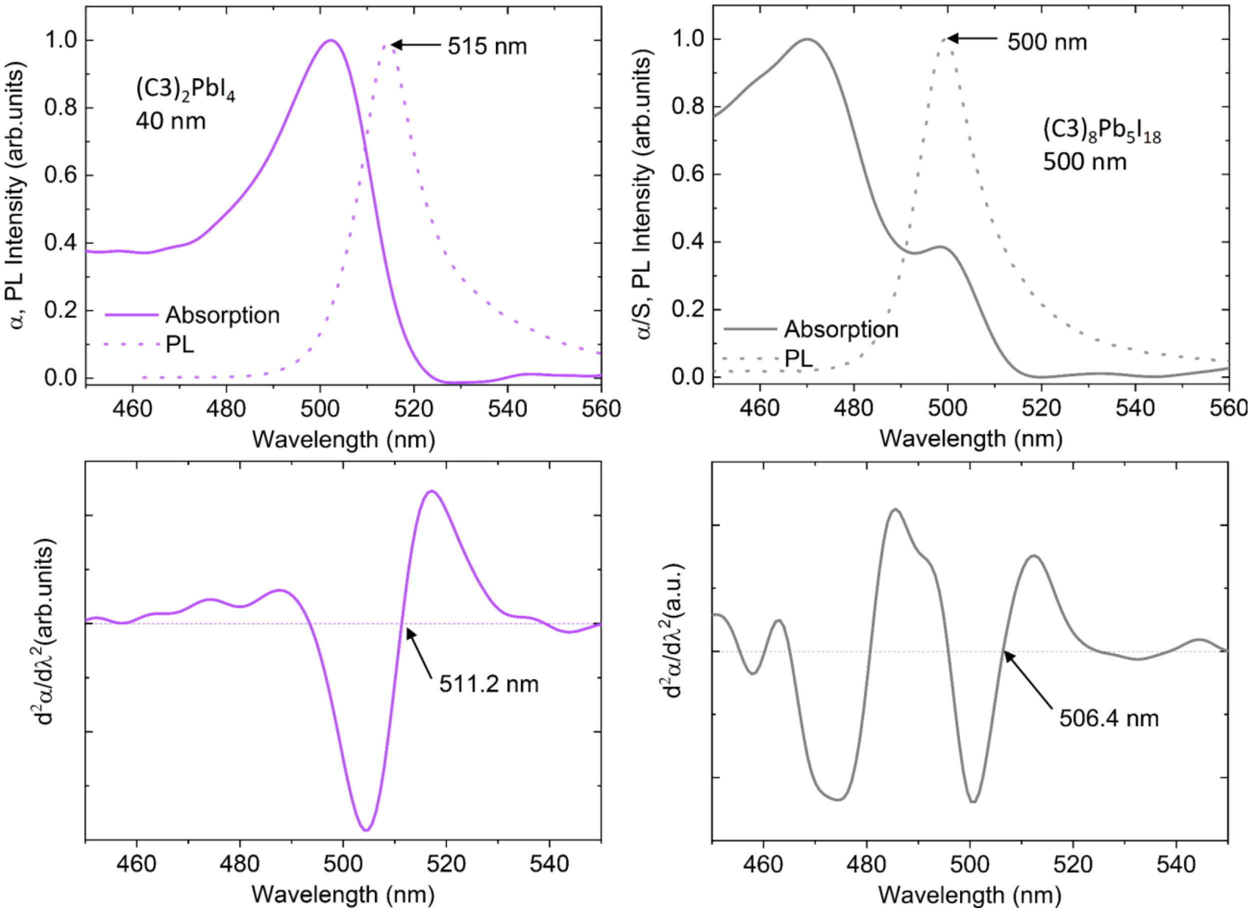

Figure S16. Normalized UV-Vis absorption and PL spectra of $(C3)_2PbI_4$ (0.05 mol/l precursor solution) and $(C3)_8Pb_5I_{18}$ (1.0 mol/l precursor solution). The absorption spectrum of $(C3)_8Pb_5I_{18}$ is determined by the diffuse reflectance due to its opacity in the spectral range of the setup. In contrast, $(C3)_2PbI_4$, prepared as a much thinner film to maintain the desired polymorph, was measured by combining reflectance and transmission. The change in thickness and absorption technique is capable to give a fingerprint on the change of absorption onset, but the quantitative band gap values should be interpreted cautiously in comparison to other spacers. However, PL measurements were performed under identical conditions for both polymorphs, revealing a higher band gap for $(C3)_8Pb_5I_{18}$.

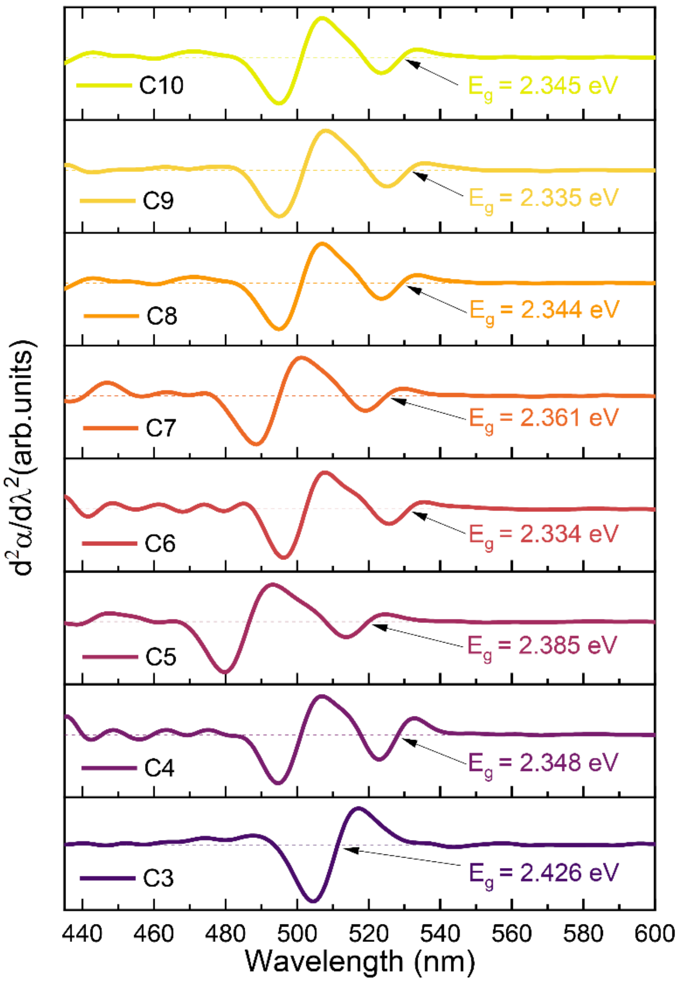

Figure S17. Second derivative the UV-Vis absorption spectra (shown in Figure 3a of the main article) which are employed to determine the inflection point of the absorption spectra which is used as the energy gap E_g of the respective material in Figure 3b.

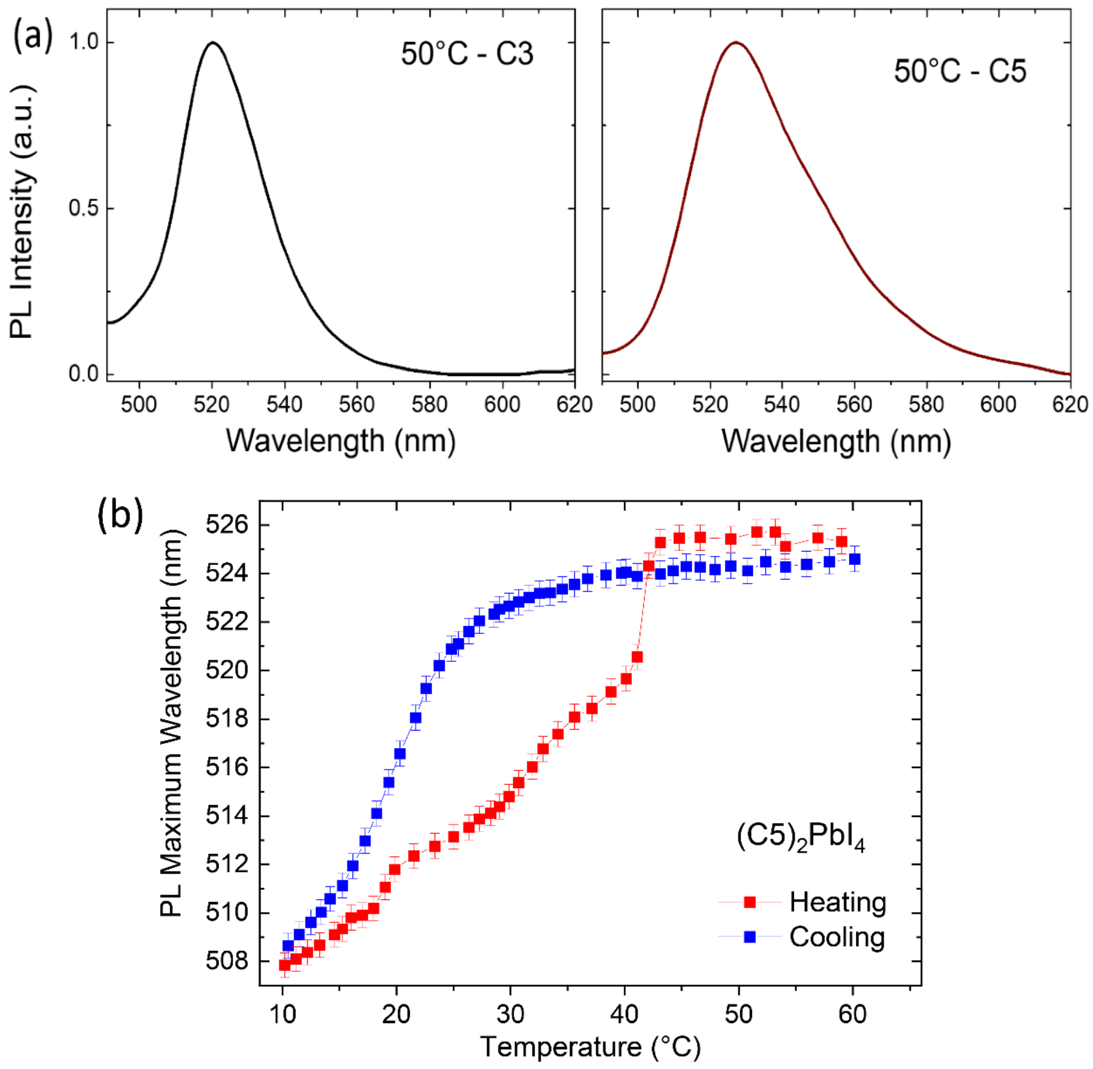

Figure S18. Investigation of optical properties at elevated temperatures (a) PL spectra of C3 and C5 at 50°C. (b) Temperature dependent PL peak position of C5. The PL maximum is red-shifting continuously upon heating, with the most pronounced change occurring at 40°C, which is attributed to the phase transition temperature (Ref. 2). Note, the shift in PL maxima by the phase transition is reversible, however it shows a hysteresis. Intriguingly, the PL maxima hysteresis aligns with the temperature-dependent changes in lattice spacing reported in literature (Ref. 7). The hysteresis, also observed in thermal investigation, is characteristic of phase transitions in the studied 2D perovskite compounds (Refs.1,2). Despite these observations, the fundamental origin of this phenomenon remains unclear and is not yet fully addressed in the literature.

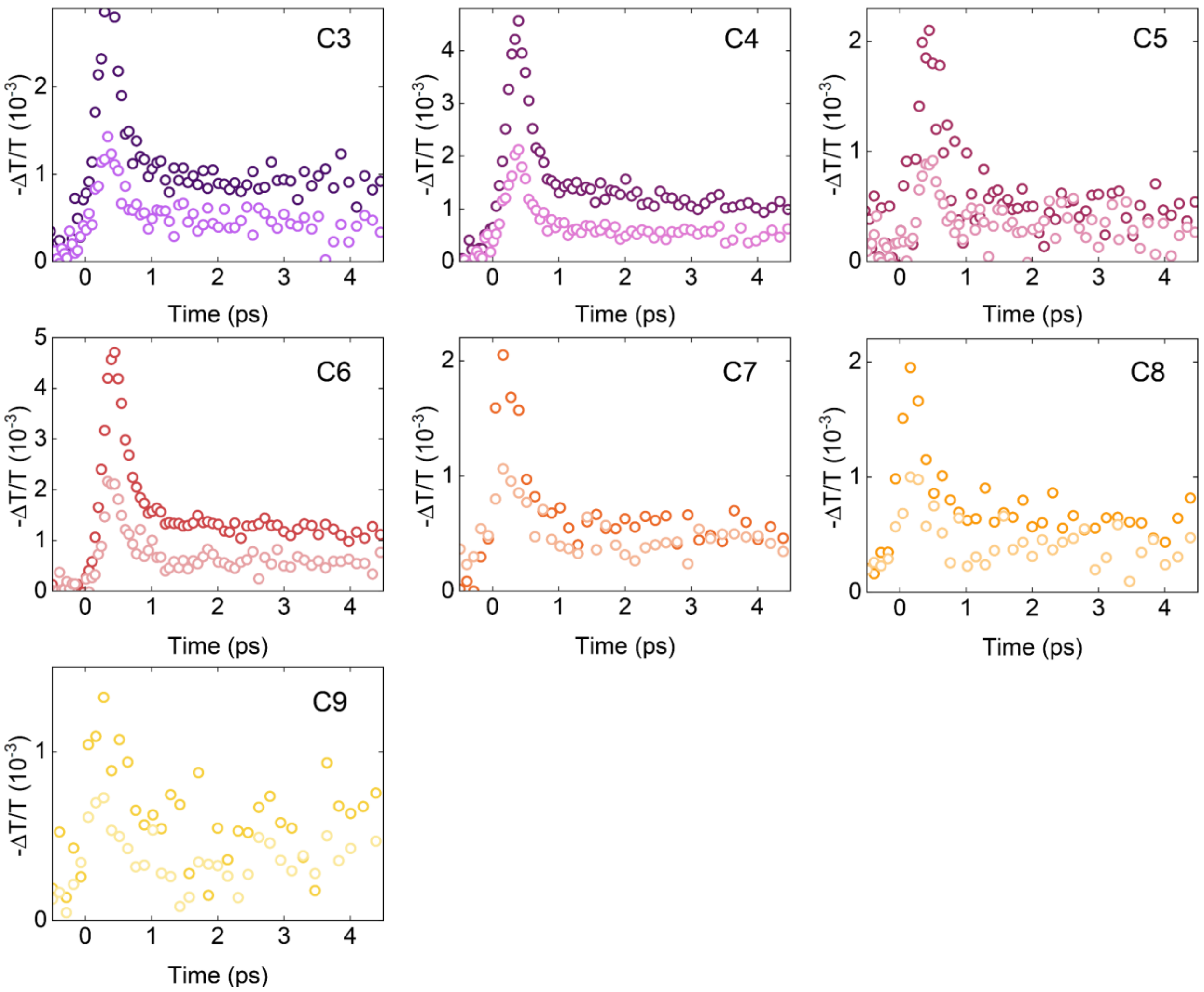

Figure S19. Fluence-dependent OPTP transients for the C3 - C9 spacer series. The 2D perovskites thin films were excited with high-energy (3.1 eV) laser pulses with excitation fluence varied between 21 μJ cm^{-2} (dark-coloured circles) and 9 μJ cm^{-2} (light-coloured circles). The spacer chain length is indicated in each panel.

From these measurements, the effective charge-carrier mobility can be estimated following the method proposed by Wehrenfennig et al. (Ref. 8) and explained in greater detail by Xia et al. (Ref. 9). For thin films with a thickness t smaller than the wavelength λ_{THz} of the incident THz radiation $t \ll \lambda_{THz}$, the fractional change in the transmitted THz electric field $\Delta T/T$ is proportional to the sheet photoconductivity. Thus, for a semiconductor thin film on a quartz substrate the resulting sheet photoconductivity can be expressed as

$$\Delta S = -\epsilon_0 c(n_{quartz} + n_{vacuum})\left(\frac{\Delta T}{T}\right) \quad \text{(Eq. 1)}$$

where n_{quartz}= 2.13 and n_{vacuum} = 1 are the refractive indexes of quartz and vacuum, respectively.[10] The effective charge-carrier mobility can be derived from the sheet photoconductivity. To do so, the number of charge carriers initially generated in the semiconductor film from the visible photoexcitation pulse must be estimated as

$$N = \varphi \frac{E}{\varepsilon}\left(1 - R_{pump} - T_{pump}\right) \quad \text{(Eq. 2)}$$

Where φ is the photon-to-charge branching ratio (i.e., the fraction of generated charges per absorbed photon absorbed), E is the pump pulse energy, ε is the excitation energy, and R_{pump} and T_{pump} are the reflectance and transmittance of the sample at the excitation energy (3.1 eV, 400 nm). Here, according to the definition of photoconductivity, Equation 1 and Equation 2 can be combined to define the charge-carrier mobility μ through the relation

$$\mu = \frac{\Delta S\, A_{eff}}{Ne} \quad \text{(Eq. 3)}$$

Where A_{eff} is the effective overlap area between THz and pump beam and e is the elementary charge. Therefore, the resulting effective charge-carrier mobility is:

$$\varphi\mu = -\epsilon_0 c(n_{quartz} + n_{vacuum}) \frac{A_{eff}\varepsilon}{eE(1 - R_{pump} - T_{pump})}\left(\frac{\Delta T}{T}\right) \quad \text{(Eq. 4)}$$

We note that charge-carrier mobilities extracted from OPTP measurements are effective mobilities – i.e., multiplied by the photon-branching ratio φ. This ratio varies between 0 and 1 and represent the fraction of initially generated free charge carriers. In free charge carrier materials, such as 3D perovskites, φ can be safely approximated to 1 and the effective mobility represents the charge-carrier mobility. Conversely, in excitonic materials, such as 2D perovskites, φ can be lower than 1 owing to the strong Coulomb interactions in the materials, and therefore, the effective mobility represents a lower boundary of charge-carrier mobility in the material.

Additional References

1. A. Lemmerer and D. G. Billing, *Dalton Trans.*, 2012, **41**, 1146–1157.
2. D. G. Billing and A. Lemmerer, *Acta Crystallogr., Sect. B*, 2007, **63**, 735–747.
3. S. Tao, I. Schmidt, G. Brocks, J. Jiang, I. Tranca, K. Meerholz and S. Olthof, *Nat. Commun.*, 2019, **10**, 2560.
4. S. Olthof, R. Meerheim, M. Schober and K. Leo, Phys. Rev. B, 2009, **79**, 245308.
5. S. Olthof, *APL Mater*, 2016, **4**, 091502.
6. R. W. Frei, D. E. Ryan and V. T. Lieu, *Can. J. Chem.*, 2011, **44**, 1945–1950.
7. R. L. Kingsford, S. R. Jackson, L. C. Bloxham and C. G. Bischak, *J. Am. Chem. Soc.*, 2023, **145**, 11773–11780.
8. C. Wehrenfennig, G. E. Eperon, M. B. Johnston, H. J. Snaith and L. M. Herz, *Adv. Mater.*, 2014, **26**, 1584–1589.
9. C. Q. Xia, J. Peng, S. Poncé, J. B. Patel, A. D. Wright, T. W. Crothers, M. Uller Rothmann, J. Borchert, R. L. Milot, H. Kraus, Q. Lin, F. Giustino, L. M. Herz and M. B. Johnston, *J. Phys. Chem. Lett.*, 2021, **12**, 3607–3617.
10. H. J. Joyce, J. L. Boland, C. L. Davies, S. A. Baig and M. B. Johnston, *Semicond. Sci. Technol.*, 2016, **31**, 103003.
